# Crag Is a GEF for Rab11 Required for Rhodopsin Trafficking and Maintenance of Adult Photoreceptor Cells

**DOI:** 10.1371/journal.pbio.1001438

**Published:** 2012-12-04

**Authors:** Bo Xiong, Vafa Bayat, Manish Jaiswal, Ke Zhang, Hector Sandoval, Wu-Lin Charng, Tongchao Li, Gabriela David, Lita Duraine, Yong-Qi Lin, G. Gregory Neely, Shinya Yamamoto, Hugo J. Bellen

**Affiliations:** 1 Program in Developmental Biology, Baylor College of Medicine, Houston, Texas, United States of America; 2 Medical Scientist Training Program, Baylor College of Medicine, Houston, Texas, United States of America; 3 Howard Hughes Medical Institute, Baylor College of Medicine, Houston, Texas, United States of America; 4 Program in Structural and Computational Biology and Molecular Biophysics, Baylor College of Medicine, Houston, Texas, United States of America; 5 Department of Molecular and Human Genetics, Baylor College of Medicine, Houston, Texas, United States of America; 6 Neuroscience Program, Garvan Institute of Medical Research, Sydney, New South Wales, Australia; 7 Department of Neuroscience, Baylor College of Medicine, Houston, Texas, United States of America; 8 Neurological Research Institute, Baylor College of Medicine, Houston, Texas, United States of America; New York University, United States of America

## Abstract

Rhodopsins (Rhs) are light sensors, and Rh1 is the major Rh in the *Drosophila* photoreceptor rhabdomere membrane. Upon photoactivation, a fraction of Rh1 is internalized and degraded, but it remains unclear how the rhabdomeric Rh1 pool is replenished and what molecular players are involved. Here, we show that Crag, a DENN protein, is a guanine nucleotide exchange factor for Rab11 that is required for the homeostasis of Rh1 upon light exposure. The absence of Crag causes a light-induced accumulation of cytoplasmic Rh1, and loss of *Crag* or *Rab11* leads to a similar photoreceptor degeneration in adult flies. Furthermore, the defects associated with loss of *Crag* can be partially rescued with a constitutive active form of Rab11. We propose that upon light stimulation, Crag is required for trafficking of Rh from the trans-Golgi network to rhabdomere membranes via a Rab11-dependent vesicular transport.

## Abstract 2

Transport of newly synthesized Rhodopsin upon light stimulation in adult *Drosophila* photoreceptors is mediated by a Crag/Rab11-dependent vesicular trafficking process.

## Author Summary

Animals sense light through receptors called Rhodopsins. These proteins are typically localized to stacked membranes in photoreceptors. In flies, upon light exposure, Rhodopsin undergoes conformational changes and becomes active as metarhodopsin. Metarhodopsin then initiates a signaling cascade that activates the photoreceptor cell. To deactivate the light response, metarhodopsin is converted back into Rhodopsin by absorption of another photon of light. Under certain conditions, metarhodopsin cannot be converted back to Rhodopsin, and it is then endocytosed and degraded. Rhodopsin then needs to be synthesized and delivered back to the membrane stacks. Here, we show that the Calmodulin-binding protein Crag is required for the delivery of newly made Rhodopsin to the membrane stacks. Loss of *Crag* leads to the accumulation of Rhodopsin in the cytosol, followed by shrinkage of membrane stack volume, and, eventually, photoreceptor cell degeneration. We also show that Crag activates a target protein, Rab11, which mediates the vesicular transport of Rhodospin to the membrane. Finally, we document that the human homolog of Crag, DENND4A, is able to rescue the loss of *Crag* in flies, suggesting that DENND4A functions in a similar process in vertebrates.

## Introduction

The *Drosophila* phototransduction pathway has been extensively studied [1], and most known members of the pathway were isolated in forward genetics screens by means of electroretinogram (ERG) and phototactic assays [2]–[6]. Since vision is not an essential sense, previous screens were performed on homozygous viable mutants. This strategy was highly successful and led to the characterization of numerous proteins that play a critical role in light responses. However, the phototransduction cascade is likely to also rely on components that are shared with other processes that are essential for viability. We therefore initiated a large mosaic ERG screen of lethal mutations on the X chromosome. Here, we report the characterization of *Calmodulin-binding protein related to a Rab3 GDP/GTP exchange protein* (*Crag*).

The *Crag* gene was first identified in a biochemical screen for photoreceptor Calmodulin (CaM)–binding proteins [7]. Crag contains a CaM binding site and interacts with CaM in a calcium-dependent manner [7],[8]. Mutations in the *Crag* gene were later shown to affect the epithelial architecture and polarized localization of basement membrane proteins including Perlecan, Laminin, and Collagen IV [8]. Based on sequence homology, the N-terminus of Crag contains three conserved domains: uDENN, DENN, and dDENN, and belongs to the DENN (differentially expressed in neoplastic and normal cells) protein superfamily [9]. The first DENN family member was identified in a screen for variable mRNA expression in neoplastic cells [10], and 18 genes encoding DENN domain proteins are present in the human genome. DENND4A, DENND4B, and DENND4C are the human homologs of Crag, and their cellular function in mammalian systems has not been characterized. Many DENN-domain-containing proteins have been found to interact directly and function as guanine nucleotide exchange factors (GEFs) of various Rab proteins [11]. The DENN/MADD protein was found to be a GEF for Rab3 and Rab27 [12],[13], whereas Connecdenn was found to be a GEF for Rab35 [14]. Furthermore, a genome-wide survey revealed that most DENN proteins are GEFs [15]. However, none of the DENN proteins were identified as GEFs for Rab11.

Rabs are localized to distinct intracellular membranes [16],[17], switch between the inactive (GDP-bound) and active (GTP-bound) conformational state, and respond to various signaling cues. In the active state, Rab proteins interact with their effectors and regulate vesicle trafficking at numerous different steps [16]. GEFs bind to inactive Rabs and facilitate the exchange of GDP for GTP, thereby activating the Rabs.

Rab11 has been shown to affect many cellular processes. It mediates protein recycling by regulating membrane transport from recycling endosomes [18]. It is present in the trans-Golgi network (TGN) and post-Golgi vesicles, where it is required for membrane transport from the TGN to the plasma membrane [19]. In polarized MDCK cells, Rab11 is required for apical recycling and basolateral-to-apical transcytosis of immunoglobulin receptors [20]–[22]. In motile cells, Rab11 is required for transport of integrin to the leading edge [23]. During cellularization of *Drosophila* embryos, Rab11 is required for basolateral membrane growth [24]. Rab11 has been shown to bind to a subunit of the exocyst complex, Sec15, which regulates polarized vesicle transport in epithelial cells and neurons [25]–[27]. However, despite Rab11's important cellular functions, no GEF for Rab11 has been identified to date.

Rhodopsins (Rhs) are light sensors in *Drosophila* and vertebrate photoreceptor cells. Rh1 is the major Rh in *Drosophila* and is present in R1–R6 photoreceptor cells. Upon absorption of a photon (580 nm), Rh1 undergoes a conformational change to an active form, metarhodopsin (metaRh), which in turn signals through a G-protein-coupled cascade that triggers the opening of the transient receptor potential (TRP) channel and leads to the depolarization of photoreceptor cells [1]. Besides its sensory role, Rh1 is required to form a rhabdomere terminal web, a meshwork of F-Actin cables, which is proposed to play a supporting role in the highly stacked rhabdomeric membranes [28],[29]. A complete loss of Rh1 causes a collapse of the rhabdomere membrane at ∼90% of pupal development [30].

During development of photoreceptors, Rh1 is synthesized and matures in the endoplasmic reticulum, after which it is transported to the rhabdomeres via the Golgi. Impairment of its maturation process leads to severe photoreceptor degeneration [31],[32]. Rab11 has been shown to be required for the post-Golgi trafficking of Rh1 to the apical rhabdomere membrane during the development of the photoreceptors. Rab11 colocalizes with Rh1 in the sub-rhabdomere region in vesicles, and reducing Rab11 activity causes defects in rhabdomere morphogenesis and accumulation of Rh1-positive vesicles in the cytosol [33],[34]. However, a role for Rab11 in adult photoreceptor cells and the phototransduction pathway has not been documented.

In adult flies, the conversion of metaRh1 back to Rh1 upon light exposure mainly occurs on the rhabdomere membrane upon absorption of a second photon (580 nm) [35]. In addition, some Rh1 is endocytosed and degraded through a lysosomal pathway, to scavenge spontaneously activated or phosphorylated metaRh, thereby preventing photoreceptor degeneration [34],[36]. As a consequence, newly synthesized Rh1 is delivered back to rhabdomeres to maintain Rh1 homeostasis as well as the overall rhabdomere morphology. However, it is unclear how this process is regulated in response to light exposure. Our data indicate that Crag and Rab11 play an essential role in the regulated transport of Rh1 to the rhabdomere membrane upon light stimulation and Ca^2+^ influx.

## Results

### Mutations in *XE10* Cause ERG Defects and Affect the *Crag* Gene

**Figure 1:**
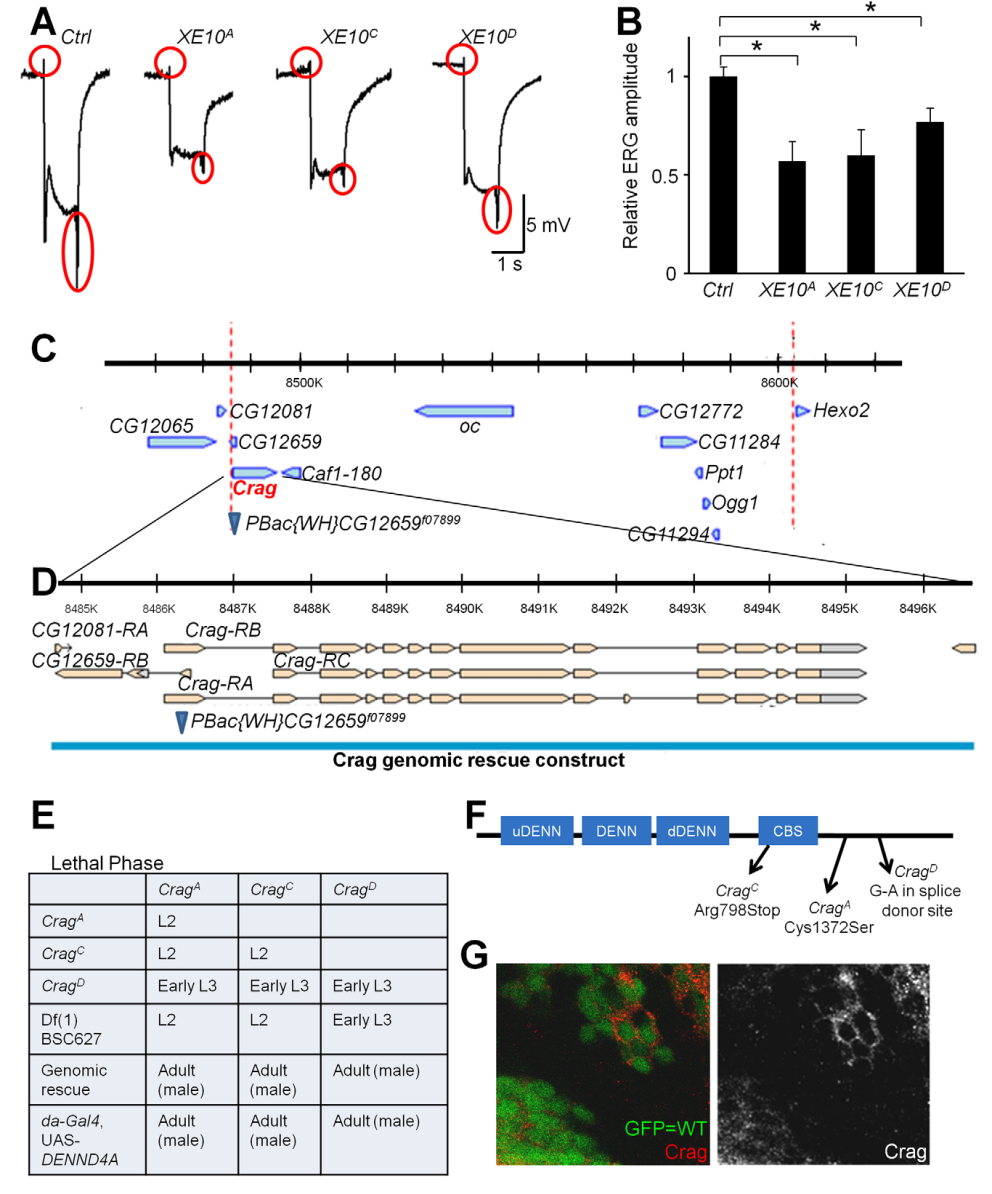
*XE10* mutant photoreceptors exhibit abnormal ERG, and *XE10* corresponds to *Crag*. (A) Representative ERG traces of 3-wk-old wild-type control flies (Ctrl; *y w FRT19A^iso^*) and flies with *XE10* mutant clones. “On” and “off” transients are marked by red circles. (B) Quantification and statistics of ERG amplitude. Five ERG traces were measured for each genotype. An asterisk indicates a *p*-value less than 0.05. (C) *XE10* alleles fail to complement a lethal insertion, which is inserted in the first exon of *Crag*. The red dotted lines mark the region of overlap of the deficiencies that fail to complement the *XE10* mutations. (D) Structure of the *Crag* gene. A 12-kb genomic rescue construct [8] that covers 2 kb upstream and 1.5 kb downstream of the *Crag* coding region rescues the *XE10* alleles. (E) Lethal phase analysis of *Crag* alleles. In the upper four lines, lethal phases of transheterozygous animals were tested. For genomic rescue experiment, heterozygous female flies were crossed with males bearing the genomic rescue construct. Rescued male progenies were identified by males that do not carry the *FM7c* balancer. For human DENND4A rescue, *Crag^(A, C, or D)^*, *FRT19A*/*FM7c*; *da-GAL4* flies were crossed with *UAS-DENND4A*, and the rescued male progenies were identified by loss of the balancer. (F) Molecular lesions identified in the three *Crag* alleles. (G) Eye imaginal discs containing *Crag^C^* clones stained with a Crag antibody. GFP expression (green) marks wild-type (WT) cells, and Crag (red) punctae are detected in the cytosol of wild-type cells but not in *Crag^C^* mutant cells.

To isolate novel genes that are involved in visual transduction, we performed an F3 forward genetic screen on the *Drosophila* X chromosome [37]. Mutations were induced using low concentrations (7.5–15 mM) of ethylmethane sulfonate on an FRT-containing X chromosome, and 33,887 stocks were screened for lethal mutations. A collection of 5,859 X-linked lethal mutations was established, and mutant clones in the eye were generated with *ey-FLP* [38]. We then performed ERG recordings on mutant photoreceptor cells of 3-wk-old flies and screened for aberrant ERGs. Hundreds of mutations were isolated, and rough mapping was performed through rescue of the lethality using X-chromosome duplications [39]. Mutations rescued by the same duplication were crossed inter se, and complementation groups were established. Here we report the characterization of one of these complementation groups. This complementation group, *XE10*, consists of three alleles (A, C, and D), and homozygous animals die as second or third instar larvae. ERGs of 3-wk-old *XE10* mutant eye clones exhibit a reduction in both the amplitude of depolarization and the size of “on–off” transients when compared to control flies (Figure 1A and 1B).

To identify the gene that is mutated in the *XE10* complementation group, we performed duplication and deficiency mapping [39],[40] and narrowed the candidate region to a ∼120-kb interval. We performed complementation tests between the *XE10* alleles and known lethal mutations in the region, and found a lethal insertion *PBac{WH}CG12659^f07899^* [41] inserted in the first exon of the *Crag* gene that fails to complement all three *XE10* alleles for larval lethality (Figure 1C). The *XE10* mutations also fail to complement a previously isolated null allele of *Crag, Crag^CJ101^* [8]. A *Crag* genomic construct rescues the lethality and phenotypes associated with all *XE10* mutations (blue in Figure 1D), showing that we have identified novel alleles of *Crag*. We identified a missense mutation in *Crag^A^* (C1371S), a nonsense mutation in *Crag^C^* (R798STOP), and a point mutation affecting a splice donor site in *Crag^D^* (Figure 1F). Lethal phase analyses indicate that the A and C alleles are severe loss-of-function or null alleles, whereas D is a hypomorphic allele (Figure 1E). We observe no immunoreactivity in *Crag^C^* clones of L3 larval eye imaginal discs using an antibody recognizing Crag, whereas in wild-type cells, the antibody reveals cytoplasmic punctae (Figure 1G). Crag is a homolog of the human DENND4A, DENND4B, and DENND4C proteins. Expression of a UAS–human DENND4A construct using the ubiquitous *daughterless-GAL4* driver rescues the lethality caused by loss of *Crag* (Figure 1E), indicating that Crag and DENND4A have conserved functions.

### *Crag* Mutant Photoreceptors Exhibit Activity-Dependent Photoreceptor Degeneration

**Figure 2:**
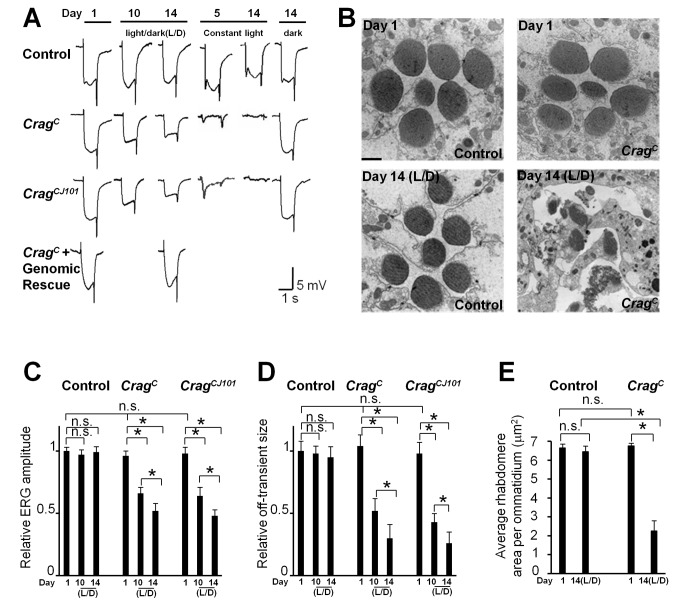
Mutations in *Crag* lead to a light-dependent photoreceptor degeneration. (A) ERG traces of wild-type control (*y w FRT19A^iso^*) and *Crag* mutant clones in flies raised in a 12-h on/off light cycle (L/D), or in constant light, or in the dark at different ages. Note that both the depolarization amplitude and the size of “on” and “off” transients become smaller upon light/dark exposure in *Crag* mutants but are not affected in wild-type controls. Constant light triggers a more severe reduction of ERG amplitude in *Crag* mutant cells. These defects are strictly light-dependent and can be rescued by the genomic rescue construct. (B) TEM of ommatidia cross-sections. Rhabdomere structures are recognized by their high electron density. Note that the aged *Crag* mutant photoreceptor cells are largely disrupted (lower right). Scale bar, 1 µM. (C) Quantification of ERG depolarization amplitudes shown in (A). Ten ERG traces were measured for each genotype at each time point. An asterisk indicates a *p*-value less than 0.05; “n.s.” indicates a *p*-value greater than 0.05. (D) Quantification of ERG off-transient amplitudes shown in (A). Ten ERG traces were measured for each genotype at each time point. (E) Quantification of rhabdomere areas shown in (C). Rhabdomere area in *Crag* mutant photoreceptors is largely reduced after 2 wk of light exposure. The rhabdomeres were outlined and their sizes were calculated using Image J software. Ten ommatidia from different cross-sections were analyzed for each genotype.

Since *Crag* mutant clones exhibit reduced ERG amplitude in 3-wk-old flies, we first examined whether the development of the visual system is affected by *Crag* mutations. To assess the morphology of photoreceptors, we performed cross-section and transmission electron microscopy (TEM) analysis of the eyes of newly eclosed flies. The data show that *Crag* mutant photoreceptors have normal rhabdomere morphology at day 1 (Figure 2B), indicating that the photoreceptors develop properly. To examine axonal targeting of the photoreceptors, we immunohistochemically stained the terminals of R7 and R8 in the medulla for Chaoptin, a photoreceptor-membrane-specific protein [42]. As shown in Figure S1, the two-layer projection patterns of R7 and R8 photoreceptors in the medulla in control and *Crag* mutant cells are indistinguishable. We then performed TEM in the lamina to determine whether R1–R6 photoreceptors target properly. *Crag* mutant photoreceptors form cartridges with the normal complement of photoreceptor terminals and synapses (Figure S2A–S2D). These data indicate that the photoreceptors display proper axonal guidance and synapse formation. Hence, the aberrant ERGs of *Crag* mutant clones are not likely due to developmental defects.

To determine whether *Crag* mutations cause photoreceptor degeneration, flies were kept either in a 12-h on/off light (∼1,800 Lux) cycle or in constant darkness, and ERGs were recorded at different time points. Flies carrying eye-specific clones of the *Crag^C^* or *Crag^CJ101^* null alleles (Figure S3A and S3B) exhibit normal ERG responses at day 1 when kept in the dark, indicating that the photoreceptors develop and function properly (Figure 2A, 2C, and 2D). To further examine whether phototransduction is affected by *Crag* mutations in dark-raised flies, we performed intracellular recording of single photoreceptor cells. The data show that *Crag* mutant photoreceptors exhibit normal response to light stimulation when compare to controls (Figure S3C–S3E).

However, both the ERG amplitude and the size of on–off transients become smaller with age when the flies are reared in a 12-h on/off light cycle, whereas the ERGs of wild-type controls are unaffected in aged flies (Figure 2A, 2C, and 2D). Interestingly, *Crag* mutant clones still exhibit a normal ERG response when kept in the dark for 2 wk. The phenotypes of aged flies kept in a 12-h on/off light cycle are fully rescued by the genomic rescue construct (Figure 2A). Moreover, when the flies are exposed to constant light, ERG amplitudes of *Crag* mutant clones are severely affected, and a 90% reduction of ERG amplitude is observed in *Crag* clones after only 5 d. After 2 wk in constant light, the ERG responses are completely abolished in *Crag* mutant cells (Figure 2A). Since Rh1 is the major light sensor of *Drosophila* photoreceptors, we also measured the Rh1 levels of flies exposed to light for different periods of time (Figure S4). The data show that the Rh1 levels of *Crag* mutant clones gradually decrease when the flies are aged in a light/dark cycle but not when they are kept in the dark. These data show that mutations in *Crag* cause a light- and age-dependent disruption of photoreceptor cell function.

To determine whether the photoreceptor cells undergo neurodegeneration, we performed cross-section and TEM analysis of the fly eye. *Crag* mutant photoreceptors have normal rhabdomere morphology in newly eclosed animals, but upon 2 wk of light exposure, the rhabdomeres become severely damaged (Figure 2B and 2E). Since the sizes of the on–off transients are reduced in *Crag* mutant cells upon light exposure, we also examined the morphology of the photoreceptor terminals after 14 d of incubation in the light/dark cycle. The morphology of the terminals is also disrupted in *Crag* mutant clones, and subcellular organelles such as capitate projections, mitochondria, and active zones are barely recognizable (Figure S2F). The wild-type controls, as well as mutant flies that carry a genomic rescue construct (data not shown), maintain a normal morphology of photoreceptors upon light exposure (Figures 2B, 2E, and S2E). These data show that *Crag* mutations indeed cause light-induced photoreceptor cell degeneration.

### Mutations in *Crag* Cause Cytoplasmic Rh1 Accumulation in Photoreceptors upon Light Stimulation

**Figure 3:**
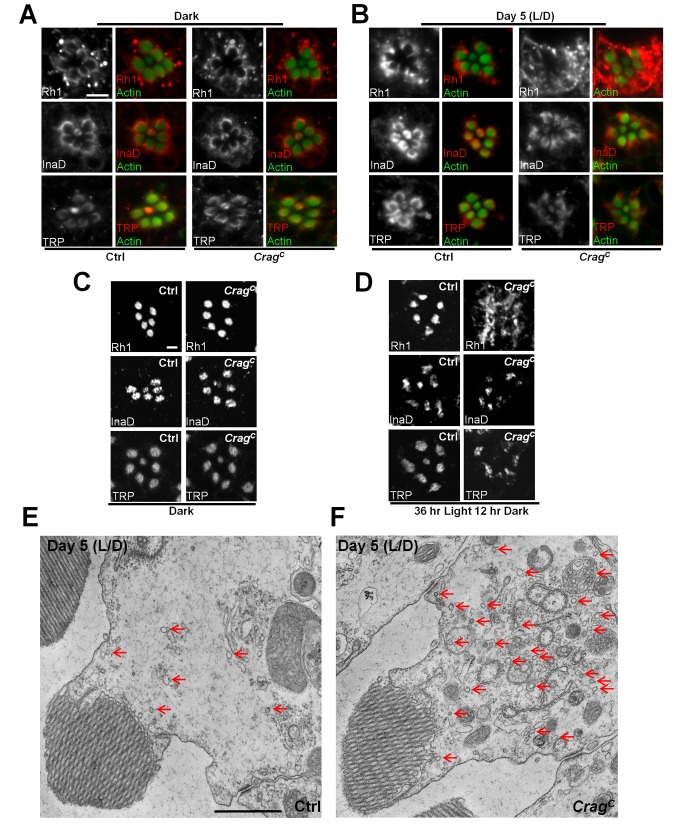
Rh1 accumulates in the cytosol of *Crag* mutant photoreceptor cells upon light stimulation. (A) Whole mount staining of photoreceptors of flies raised in the dark. Note that Rh1, InaD, and TRP all exhibit crescent shaped patterns at the base of the rhabdomeres in both genotypes. Rhabdomeres are labeled by phalloidin staining of Actin. Scale bar, 5 µM. (B) Same staining as (A) in control (Ctrl) and *Crag* mutant flies upon 5 d of light/dark (L/D) exposure. Note the cytosolic accumulation of Rh1 in *Crag* mutant photoreceptors. (C) Cross-sections of control (*y w FRT19A^iso^*) and *Crag* mutant photoreceptors in flies raised in dark were stained with antibody against Rh1, InaD, and TRP. All markers examined exhibit a normal distribution in both genotypes. Note that all the proteins are uniformly distributed in the rhabdomeres. Scale bar, 2 µM. (D) Same staining as (C) for flies exposed to 36 h of light and then kept for 12 h in the dark. Note that Rh1, but not InaD and TRP, is accumulated in the cytosol in *Crag* mutants. (E and F) TEM of a photoreceptor cross-section of a control and a *Crag* mutant photoreceptor upon 5 d of light/dark exposure. Note the accumulation of numerous vesicles (red arrows) in *Crag* mutant cells. Scale bar, 1 µM.

Since loss of *Crag* causes a light-dependent degeneration, we hypothesized that Crag in photoreceptor cells may play a role in phototransduction. Upon light stimulation, Rh1 undergoes a conformational change to metaRh, which consequently triggers the opening of TRP channels through G-protein-coupled signaling and leads to the depolarization of photoreceptor cells [1]. InaD contains five PDZ domains and serves as a scaffold protein to allow many players in the pathway to form a signaling complex [43]–[45]. We performed immunostainings of major phototransduction proteins with a whole mount protocol to assess whether *Crag* is required for their subcellular distribution. Rh1, TRP, and InaD are all properly localized in *Crag* mutant photoreceptors in flies kept in dark, as the staining patterns are similar to those of wild-type cells (Figure 3A). These data are in agreement with the functional data, as *Crag* mutant clones exhibit normal ERG responses in newly eclosed flies or flies kept in darkness for several weeks. Upon 5 d of exposure to a light/dark cycle, these proteins are still properly localized in control photoreceptors. However, in *Crag* mutant cells, Rh1 is massively accumulated in the cytosol (Figure 3B). In contrast, a cytosolic accumulation of TRP and InaD is not observed (Figure 3B). Notably, with the whole mount staining protocol, a crescent shaped distribution pattern of these proteins is observed at the base of the rhabdomeres, which is consistent with previous studies [36],[46]–[48]. In contrast, a uniform distribution of these proteins in the rhabdomeres has been observed in immunostainings of thin sections [49]–[52]. We therefore also performed immunostainings in cross-sections of photoreceptors and observed a rhabdomeric localization of Rh1, InaD, and TRP in flies kept in dark (Figure 3C). Similarly, the data also show that light stimulation induces a cytosolic accumulation of Rh1, but not TRP and InaD, in *Crag* mutant cells (Figure 3D). The difference in localization between the two protocols is probably due to a different accessibility of the antibodies, since rhabdomeres are highly packed membrane stacks. These data suggest that *Crag* is involved in Rh1 transport upon light stimulation but not during photoreceptor development.

Since Rh1 is a transmembrane protein, its accumulation in the cytosol of *Crag* mutant cells upon light stimulation would imply an accumulation of membrane structures. We therefore examined the ultrastructure of the light-stimulated photoreceptor cells by TEM. After 5 d of 12-h on/off light stimulation, *Crag* mutant cells exhibit a massive accumulation of vesicles in the cytosol (25.2±8.4 in wild type; 186.8±21.3 in *Crag* mutants: *n* = 10, *p*<0.001) when compared to controls (Figure 3E and 3F). The accumulation of Rh1 as well as vesicles in the cytosol indicates a defect in vesicular trafficking of Rh1 in *Crag* mutant cells.

### Crag Is Required for the Transport of Newly Synthesized Rh1 to the Rhabdomeres during Light Stimulation

**Figure 4:**
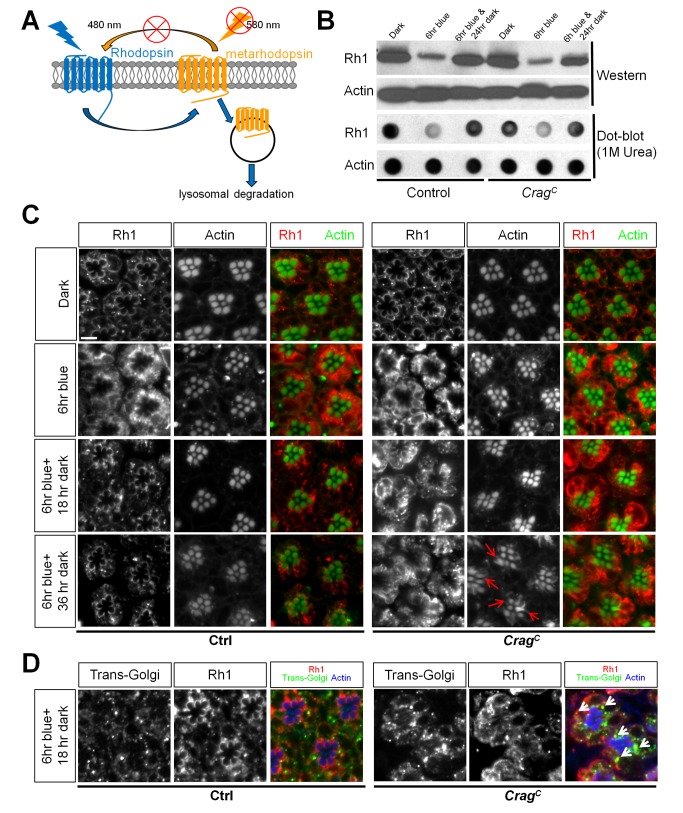
Crag is required for Rh1 transport to the rhabdomeres upon light stimulation. (A) In adult flies, blue light triggers the conformational change of Rh to metaRh, whereas orange light is required for converting metaRh to Rh. In the presence of blue light and absence of orange light, metaRh is accumulated in the rhabdomere, which in turn triggers massive endocytosis and degradation of Rh. (B) Fly heads with control or *Crag^C^* mutant clones in the eyes were collected from flies that were kept in the dark or in blue light for 6 h with or without 24 h of recovery. Western blots and dot blots were performed to detect total Rh1 and Actin (loading control) levels. Note that in both genotypes, the Rh1 level significantly decreased after 6 h of blue light treatment and was restored after 24 h of recovery in the dark. (C) Whole mount staining of Rh1 in photoreceptors kept in the dark, exposed to 6 h of blue light, or exposed to 6 h blue light with 18 or 36 h of recovery in the dark. 6 h of blue light treatment triggers massive endocytosis of Rh1 in both control and *Crag* mutant photoreceptors. Note the strong Rh1 staining in the cytosol. After 18 h of recovery, a crescent shaped pattern of Rh1 is reformed in controls but not in *Crag* mutant photoreceptors. Rh1 accumulation is persistent in *Crag* mutant cells after 36 h of recovery. Note that breakdown of rhabdomeres (indicated by red arrows) is observed in flies kept for 36 h in the dark but not in flies kept for 18 h in the dark after blue light exposure. Scale bar, 5 µM. (D) Immunostaining of the trans-Golgi compartment (Peanut agglutinin) and Rh1 in flies after 6 h of blue light exposure and 18 h of dark recovery. Note the enlargement of the trans-Golgi compartment and the colocalization between Rh1 and the trans-Golgi compartment (indicated by white arrowheads) in *Crag* mutant cells.

During photoreceptor development, Rh1 is delivered into rhabdomeres through a Rab11-mediated vesicle transport [34]. In adult flies, a subpopulation of metaRh is endocytosed and degraded [36],[46], and this Rh1 loss should be replenished with newly synthesized Rh1 to maintain homeostasis. The accumulation of vesicles in *Crag* mutant photoreceptors could be due to an increase in endocytosis, a decrease in the clearance of the endocytosed vesicles, or a failure to secrete newly synthesized vesicles. With white light stimulation, Rh1 is internalized at a very slow rate, as determined by Western blots and immunostainings (Figure S5A and S5B). Therefore, we kept flies for a 6-h period in blue light to determine which aspect of Rh1 trafficking is affected by *Crag*. Blue light converts Rh1 to metaRh, and metaRh requires orange light (580 nm) to be converted back into Rh1. In the absence of orange light, blue light triggers massive endocytosis and degradation of metaRh [53] (Figure 4A). Western blot data show that exposure for 6 h to blue light leads to a similar decrease of Rh1 in both wild-type and *Crag* mutant cells (Figure 4B, upper panel), indicating that endocytosis and degradation of metaRh is not significantly affected in *Crag* mutant photoreceptors. Moreover, upon 24 h of recovery in the dark, there is a significant increase in Rh1 levels in both wild-type and *Crag* mutant cells, indicating that the de novo synthesis of Rh1 is also not affected in *Crag* mutant cells. Previous studies have shown that internalized Rh1 may form insoluble aggregates that are not detectable in Western blot [36]. We therefore homogenized fly heads in SDS buffer containing 1 M urea and performed dot blots to assess total Rh1 levels. The data confirm that Rh1 is indeed degraded upon 6 h of blue light stimulation in both wild-type and *Crag* mutant cells (Figure 4B, lower panel). In summary, Crag does not appear to affect endocytosis, degradation, or synthesis of Rh1.

We further examined Rh1 dynamics upon blue light stimulation with the whole mount staining protocol. As shown in Figure 4C, 6 h of blue light stimulation triggers massive endocytosis of Rh1 in both wild-type and *Crag* mutant photoreceptors. After 18 h of recovery in the dark, cytosolic Rh1 is reduced and the crescent shaped pattern is re-formed in the wild-type photoreceptors, whereas in *Crag* mutant photoreceptors, Rh1 remains accumulated in the cytosol. Furthermore, the accumulation of Rh1 in *Crag* mutants persists even after 36 h of recovery.

Blue light exposure drives photoreceptors into prolonged depolarizing afterpotential (PDA) [35]. To test whether the accumulation of Rh1 in *Crag* mutant cells is caused by a PDA, we exposed the flies to blue light followed by orange light for 20 min to terminate the PDA. We then kept the flies for 18 h in the dark and examined the Rh1 distribution (Figure S5C). Rh1 still accumulates in the cytosol of *Crag* mutant photoreceptors but not in controls, indicating that Rh1 localization defects are not caused by PDA.

We next performed TEM analysis of photoreceptors during the course of the blue light treatment and the recovery (Figure S6). The data indicate that electron densities in the cytosol correlate with the Rh1 levels in the cytosol. Interestingly, after 36 h of dark recovery, the rhabdomeres start to break down in *Crag* mutant cells, as revealed by Actin staining and TEM analyses (Figure S6). The data suggest that persistent accumulation of Rh1 in the cytosol leads to the breakdown of rhabdomeres and the degeneration of photoreceptor cells.

Since the degradation of Rh1 is unaffected in *Crag* mutant cells, we hypothesized that the accumulation of Rh1 in the cytosol may be the result of a defect in transport of newly synthesized Rh1 to the rhabdomeres. We therefore stained the photoreceptors with a trans-Golgi marker, Peanut agglutinin [54], and observed an expansion of the TGN in *Crag* mutant photoreceptors (Figure 4D). Moreover, a portion of the accumulated Rh1 colocalizes with the trans-Golgi marker. These data, along with the vesicle accumulation observed by TEM, suggest that Crag is required for post-Golgi trafficking of Rh1 to the rhabdomeres. In summary, loss of *Crag* leads to accumulation of newly synthesized Rh1 in post-Golgi vesicles, a breakdown of rhabdomeres, and, eventually, photoreceptor degeneration.

### Knockdown of Rab11 in Adult Eyes Causes Photoreceptor Degeneration

**Figure 5:**
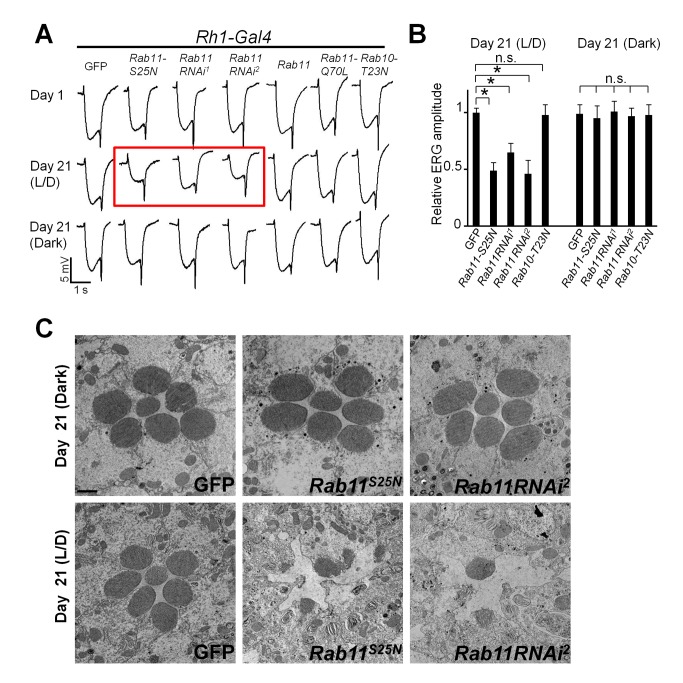
Knockdown of Rab11 in adult eye causes light-dependent photoreceptor degeneration. (A) ERG traces of flies with expression of *UAS-GFP, Rab11-DN(S25N), Rab11* double-stranded RNA constructs *(Rab11 RNAi^1^* and *Rab11 RNAi^2^), Rab11, Rab11-CA(Q70L)*, and *Rab10-DN(T23N)* driven by *Rh1-GAL4* at day 1 and day 21 with or without 12-h on/off light exposure (L/D). Note that the ERG amplitudes are reduced in the flies with Rab11 knockdown and light exposure (red box). (B) Quantification of ERG depolarization amplitudes shown in (A). Five ERG traces were measured for each genotype at each time point. An asterisk indicates a *p*-value less than 0.05; “n.s.” indicates a *p*-value greater than 0.05. (C) TEM of photoreceptor cross-sections of flies expressing GFP, Rab11-DN, or Rab11 RNAi^2^ in photoreceptor cells after 21 d of incubation in light/dark cycle or in constant dark. Note that the rhabdomere morphology is disrupted in flies with Rab11 knockdown upon light exposure. Since Rh1-GAL4 drives expression only in R1–R6 photoreceptors, R7/R8 are better preserved than R1–R6. Scale bar, 1 µM.

Crag possesses three DENN domains and may serve as a GEF for a Rab protein. To identify its potential target(s), we performed a screen using a collection of 31 Rab dominant negative (DN) transgenic lines [55] (Figure S7). To bypass the requirement of some Rabs for the development of photoreceptor cells, we used Rh1-GAL4, which is expressed late in photoreceptor formation and in adult photoreceptors, to drive expression of the Rab-DNs. To lower expression levels we also raised the flies at 18°C prior to eclosion. We then aged the flies in a 12-h light/dark cycle or darkness for 21 d and performed ERGs. Expression of only Rab11-DN (S25N) causes a light-dependent reduction of ERG amplitude in 3-wk-old flies exposed to light, similar to that in *Crag* mutants (Figures 5A, 5B, and S7). To further confirm that loss of Rab11 activity in the adult eye leads to a light-dependent photoreceptor degeneration, we expressed two Rab11 double-stranded RNA constructs [34],[56] in the adult eye using Rh1-GAL4 and found that they also cause a reduction of ERG amplitude upon light stimulation (Figure 5A and 5B). In contrast, expression of wild-type or constitutively active (CA) Rab11 does not cause a phenotype (Figure 5A and 5B). Next, we performed TEM after light and dark exposure of photoreceptors with Rab11 knockdown. Light exposure disrupts the photoreceptor cell morphology in these cells, whereas dark incubation causes no obvious effects after 3 wk (Figure 5C). These data indicate that knockdown of Rab11 leads to a light-induced photoreceptor degeneration similar to that of loss of *Crag*. Hence, Rab11 is a potential target of Crag.

### Crag Is a GEF for Rab11

**Figure 6:**
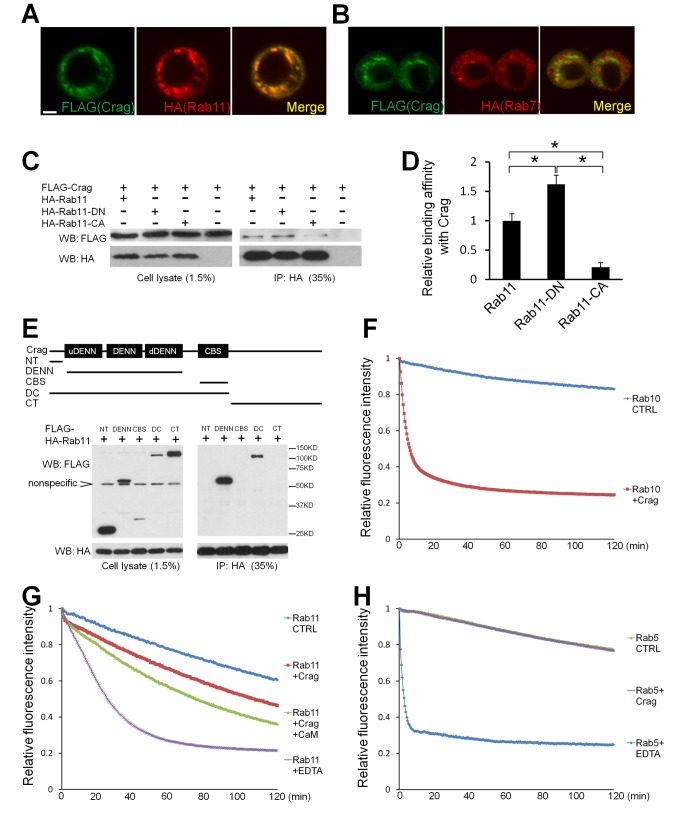
Crag interacts with Rab11 in vitro. (A) Immunostaining of S2 cells transfected with FLAG-tagged Crag (green) and HA-tagged Rab11 (red). Their expression regions largely overlap. Scale bar, 2 µM. (B) Immunostaining of S2 cells transfected with FLAG-tagged Crag and HA-tagged Rab7. No significant colocalization is observed. (C) S2 cells transfected with FLAG-tagged Crag and/or HA-tagged Rab11, Rab11-DN, or Rab11-CA were harvested and lysed. Anti-HA gel was used to pull down HA-tagged proteins. When Rab11 and Crag are co-transfected, Crag was also co-immunoprecipitated, as detected on Western blots, indicating that Crag binds to Rab11. Also note that Crag preferentially binds to the DN form of Rab11 rather than the CA form. (D) Quantification and statistics of binding affinity between Crag and the different forms of Rab11 shown in (A). The data are quantified using the Gel analysis function in Image J. Results from three independent experiments were analyzed. An asterisk indicates a *p*-value less than 0.05. (E) A scheme of different Crag deletion constructs and their binding affinity with Rab11 was tested by co-IPs. Crag segments containing DENN domains (DENN and DC) bind to Rab11, whereas the segments that lack DENN domains (NT, CBS, and CT) do not, indicating that the interaction between Crag and Rab11 is dependent on DENN domains. (F) GEF assay of Crag and Rab10. Crag and Rab10 proteins were purified using a baculovirus system. Rab10 was preloaded with fluorescence-labeled BODIPY-GDP, and then GTP was added with or without Crag. The change of fluorescence intensity was recorded for 2 h. Crag promotes the release of GDP from Rab10, indicating it possesses GEF activity against Rab10. (G) GEF assay of Crag and Rab11. Crag promotes GDP release from Rab11, and the presence of CaM and calcium further enhances the release rate. 10 mM EDTA was used as a positive control, as EDTA absorbs Mg^2+^ from Rab11 and triggers GDP release. (H) GEF assay of Crag and Rab5. No GEF activity against Rab5 was observed, whereas 10 mM EDTA triggers a rapid release of GDP from Rab5.

To assess whether Crag and Rab11 physically interact, we generated tagged Crag (FLAG) and Rab11 (HA) expression constructs and first examined their protein localizations in *Drosophila* S2 cells. Crag colocalizes with Rab11 when both are co-expressed (Figure 6A). When Rab7 and Crag are co-expressed, the large overlapping punctae observed when Rab11 and Crag are co-expressed are not obvious (Figure 6B). We then performed co-immunoprecipitation (co-IP) using an antibody against HA to pull down Rab11, and found that Crag is co-precipitated with Rab11, indicating that Crag is a binding partner of Rab11 (Figure 6C). GEFs bind to the GDP-loaded Rabs to promote the release of GDP, whereas binding of GTP to Rabs diminishes the binding of the GEFs. Hence, GEFs have a higher affinity for GDP-bound forms of Rabs than for GTP-bound forms. We therefore performed co-IP between Crag and the DN (mostly GDP-bound) or CA (mostly GTP-bound) form of Rab11. The results show that Crag binds preferentially to the Rab11-DN, and weakly to Rab11-CA (Figure 6C and 6D). We next mapped the Rab11 binding domain of Crag by generating a series of deletion constructs. The constructs containing the three DENN domains co-immunoprecipitate with Rab11, whereas those lacking DENN domains do not co-immunoprecipitate with Rab11 (Figure 6E). These data suggest that Crag may be a GEF for Rab11.

To determine whether Crag possesses GEF activity, we performed in vitro activity assays. Unfortunately, expression of the 180-kDa Crag protein in *Escherichia coli* produces insoluble protein in inclusion bodies. We therefore purified the protein using a baculovirus expression system in insect cells. For the GEF assay we preloaded the Rab proteins with fluorescence-labeled BODIPY-GDP, and then added excessive unlabeled GDP with or without the potential GEF and measured the release rate of BODIPY-GDP from the Rabs. Since the human homolog of Crag, DENND4A, was shown to exhibit GEF activity against Rab10 [15], we first performed the GEF assay with Rab10. As shown in Figure 6F, Crag strongly promotes GDP release from Rab10, indicating that Crag and DENND4A are functionally conserved and that the purified Crag protein is a GEF in vitro. However, Rab10 is not expressed in the adult eye [57], and expression of Rab10-DN does not cause a light-dependent degeneration (Figure 5). Hence, the degeneration phenotypes associated with *Crag* mutations are unlikely to be caused by defects in Rab10 activation.

Next, we performed the GEF assay for Crag against Rab11 and Rab5. As shown in Figure 6G and 6H, Crag indeed facilitates the GDP dissociation from Rab11 but not from Rab5. However, the kinetics of the reaction with Rab11 is slow compared to that of Crag against Rab10. We hypothesized that the slow kinetics is due to the molecular properties of purified Rab11. We therefore added 10 mM EDTA to GDP-preloaded Rab11 to examine its release kinetics. EDTA absorbs Mg^2+^ from the Rab proteins and induces a rapid release of GDP [58]. Although the kinetics for GDP release is significantly accelerated, it is relatively slow when compared to EDTA-triggered GDP release of Rab5 (Figure 6G and 6H). We concluded that the kinetics of Rab11 in vitro is generally slow. Since Crag binds to CaM in a calcium-dependent manner [7],[8], and since cellular Ca^2+^ levels increase during photoactivation, we next examined whether CaM regulates Crag activity. In the presence of CaM and Ca^2+^, the exchange rate is indeed increased (Figure 6G). Hence, it's possible that Crag activity is enhanced during light stimulation upon a Ca^2+^ influx in photoreceptor cells. These data, together with our in vivo observations, indicate that Crag is a GEF for Rab11.

**Figure 7:**
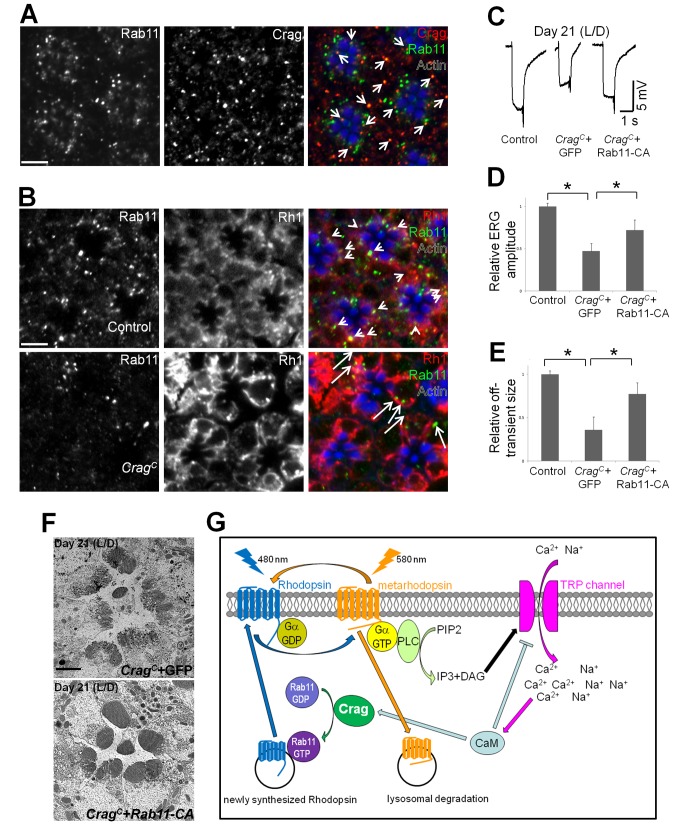
Crag genetically interacts with Rab11 in vivo. (A) Immunostaining of Crag (green) and Rab11 (red) in cross-sections of wild-type photoreceptors upon 12 h of light stimulation. The proteins colocalize in punctuate structures (indicated by arrowheads). The rhabdomeres are marked by Actin staining (blue). Scale bar, 5 µM. (B) Immunostaining of Rab11 (red) and Rh1 (green) in photoreceptors of wild-type controls and *Crag* mutants upon 12 h of light stimulation. In controls, Rab11 is present in punctae that partially colocalize with Rh1 (indicated by arrowheads), and most of the punctae are close to the rhabdomeres; in *Crag* mutants, many fewer punctae of Rab11 are observed, and these punctae are not closely associated with rhabdomeres (indicated by arrows). Scale bar, 5 µM. (C) GFP or Rab11-CA were expressed in *Crag* mutant clones, and the flies were kept in a 12 hour light/dark cycle (L/D) for 3 wk along with controls (*y w P{neoFRT}19A^iso^* with GFP expression in the photoreceptors). Representative ERG traces of these flies are shown. (D) Quantification of the ERG depolarization amplitudes shown in (B). Ten ERG traces were measured for each genotype at each time point. An asterisk indicates a *p*-value less than 0.05. (E) Quantification of the ERG off-transient amplitudes shown in (B). Ten ERG traces were measured for each genotype at each time point. (F) TEM of photoreceptor cross-sections of flies expressing GFP or Rab11-CA in *Crag* mutant photoreceptor cells after 21 d of incubation in a light/dark cycle. The morphology of the rhabdomeres is much better preserved when Rab11-CA is expressed. Scale bar, 1 µM. (G) Model of Crag function. Upon absorption of a photon, Rh1 undergoes a conformational change to the active form, metaRh, which signals through a G-protein-coupled cascade and triggers the opening of TRP channels and the influx of Ca^2+^ and Na^+^ into photoreceptor cells. MetaRh is converted back into Rh1 by exposure to another photon. However, a subpopulation of Rh1 is endocytosed and degraded through a lysosomal pathway. To replenish the Rh1 pool, Rh1 needs to be synthesized and delivered to the rhabdomeres. Rab11-mediated vesicle trafficking is required for Rh1 transport to the rhabdomeres. Crag is a GEF for Rab11 in this process, and its GEF activity maybe enhanced by CaM and Ca^2+^ influx. Hence, light stimulation not only triggers endocytosis but may also promote trafficking of Rh1 to rhabdomeres to maintain homeostasis. PLC, Phospholipase C.

We next examined the interactions between Crag and Rab11 in vivo. We first performed immunostaining of Crag and Rab11. Crag and Rab11 colocalize in punctate structures (Figure 7A), indicating that they may physically interact in vivo. The subcellular localization of Rab proteins often depends on their activation status. When bound to GTP, Rab proteins bind to various effectors that help target the proteins to the proper membrane compartments. Hence, many GEFs regulate the subcellular localization of Rabs. To determine whether Crag is required for the proper subcellular localization of Rab11, we exposed the flies to 12 h of light stimulation and performed immunostaining of Rab11 and Rh1. In control photoreceptors, Rab11 colocalizes with Rh1 in numerous punctae, and many of the punctae are in close vicinity to the rhabdomeres, providing further evidence for a role for Rab11 in regulating Rh1 transport. In *Crag* mutant photoreceptors, Rab11 exhibits a more diffuse pattern, less punctae are observed, and the punctae are rarely localized in the vicinity of rhabdomeres (Figure 7B). These data show that Crag is indeed required for the proper localization of Rab11 in adult photoreceptors.

To determine whether *Rab11* functions downstream of *Crag*, we overexpressed a CA form of Rab11 (Rab11-CA), which does not require a GEF for its activation, in *Crag* mutant cells. If Crag functions as a GEF for Rab11, expression of Rab11-CA may rescue the defects associated with the loss of *Crag*. Hence, we aged the flies for 3 wk in a 12-h light/dark cycle and examined photoreceptor function with ERGs. As shown in Figure 7C–7E, Rab11-CA expression in *Crag* mutant cells partially rescues the ERG phenotypes. We also expressed Rab10-CA in the *Crag* clones and observed no significant rescue (data not shown). To further determine whether the Rab11-CA is able to also suppress the light-induced morphological defects of photoreceptors associated with the loss of *Crag*, we performed TEM. The data show that the rhabdomeres are much better preserved upon light stimulation in *Crag* mutant cells expressing Rab11-CA than in those expressing a GFP control (Figure 7F). In addition, we compared the time course of photoreceptor degeneration using ERG recordings in flies that contained *Crag* mutant clones and/or expressed a DN mutation for *Rab11* in the eyes (Figure S8). The data show that no significant additive or synergetic effect is observed when *Crag* and *Rab11* are impaired, suggesting that they are involved in the same pathway required for photoreceptor maintenance. In summary, these data indicate that *Rab11* functions downstream of *Crag* genetically, and suggest that Crag regulates Rh1 transport via Rab11 in *Drosophila* photoreceptors.

## Discussion

Here, we show that Crag is a novel GEF for Rab11 and that it is required for the post-Golgi transport of Rh1 to the rhabdomeres during light activation (Figures 7G and S10). This regulated transport of Rh1, which is independent of Rh1 transport during the development of the photoreceptors, replenishes the loss of Rh1 induced by light stimulation. Loss of *Crag* leads to accumulation of secretory vesicles in the cytosol of photoreceptor cells, and eventually leads to a light- and age-dependent photoreceptor degeneration.

### Crag Is Required to Maintain Rh1 Homeostasis upon Light Exposure

During development of photoreceptors, Rh1 and other phototransduction proteins are synthesized in the endoplasmic reticulum and transported to the rhabdomeres to build functional photoreceptors. Some molecular players, including *Rab11* and *XPORT*, have been shown to play a role in this process [32],[34]. Upon light activation Rh1 is converted to metaRh (Figure S10A). MetaRh is then converted back into Rh1 on rhabdomere membranes via absorption of another photon, allowing the maintenance of Rh1 levels in the rhabdomere [35]. In wild-type photoreceptors, a portion of metaRh is phosphorylated and endocytosed [46], and it has been proposed that internalization of metaRh promotes the clearance of dysfunctional proteins and serves as a proofreading mechanism (Figure S10B). Internalized Rh1 is then degraded through an endosomal/lysosomal pathway [36]. Obviously, the gradual loss of Rh1 in wild-type photoreceptors leads to the necessity to constitutively synthesize Rh1 and replenish the rhabdomeric pool. This is nicely illustrated with the loss of retinol dehydrogenase (RDH), which is required for the regeneration of the chromophore of Rh1. Loss of RDH leads to progressive reduction in rhabdomere size and light-dependent photoreceptor degeneration [50].

Our data show that *Crag* is required to maintain homeostasis of Rh1 upon light stimulation. Loss of *Crag* leads to Rh1 accumulation in the cytosol and, eventually, retinal degeneration in the presence of light. Mutations in genes that affect metaRh1 turnover, such as *Calmodulin* and *arrestin* 2 [52],[59], lead to prolonged deactivation time of the photoresponse. Since both ERGs and single-cell recordings of *Crag* mutant photoreceptors are normal, it is unlikely that *Crag* is involved in the recycling of metaRh1 to Rh1. To test whether *Crag* is required for transport of newly synthesized Rh1 in adult photoreceptors, we exposed the flies to blue light to trigger massive endocytosis and degradation of Rh1, and then measured the new synthesis and transport of Rh1 back to the rhabdomeres over time. *Crag* is not required for the synthesis of Rh1. However, in *Crag* mutants, the newly synthesized Rh1 accumulates in the cytosol. We propose that Crag is required for the delivery of newly synthesized Rh1 to the rhabdomeres and that loss of *Crag* leads to a gradual reduction in the size of rhabdomeres and to degeneration of the photoreceptor cells (Figure S10C and S10D). Indeed, the time course and morphological features of degeneration associated with loss of *Crag* are very similar to the phenotypes observed in RDH mutants, further supporting that Crag is involved in the Rh1 synthesis/delivery pathway.

### Crag Is a GEF for Rab11

Rab11 has been implicated in various intracellular membrane trafficking processes. Its diverse functions in different membrane compartments are mediated through its downstream effectors in a context-specific manner; many of these functions have been identified in previous studies [60]. However, GEFs for Rab11 in any context have not yet been identified. Our in vivo and in vitro data provide compelling evidence that Crag is a GEF for Rab11. First, in *Drosophila* S2 cells, Crag colocalizes and physically interacts with Rab11. Second, Crag preferably binds to the GDP-bound form of Rab11, and the DENN domains are required for binding. Third, Crag is required for the proper localization of Rab11 in photoreceptors upon light stimulation. Fourth, loss of *Crag* or *Rab11* leads to a similar light-induced photoreceptor degeneration. Fifth, expression of Rab11-CA partially rescues the degeneration caused by *Crag* mutations. Finally, an in vitro GEF assay shows that Crag facilitates the release of GDP from Rab11. It has been previously established that Rab11 is essential for photoreceptor cell development and Rh1 transport during pupal stages [34]. However both rhabdomere morphology and Rh1 localization are normal in *Crag* clones in newly eclosed flies. Similarly, initial deposition of TRP is also not affected by *Crag* mutations, in agreement with previous findings that Rh1 and TRP are co-transported to the rhabdomeres during their development [32]. Interestingly, cytosolic localization of TRP is not observed in *Crag* mutant photoreceptor cells exposed to light, suggesting that during light stimulation, Rh1 and TRP dynamics are distinct. Indeed, internalization of TRP upon light stimulation has not been reported in previous studies. Our data therefore indicate that other GEFs must exist for Rab11 during photoreceptor development, and that Crag is specifically required for Rab11 GDP/GTP exchange during light activation in adult flies. In addition, Crag may function as a GEF for Rab10 in other processes and cells, such as polarized deposition of basement membrane proteins in follicle cells.

The biochemical assay shows that the kinetics of Crag GEF activity is slow when compared to the GEF activity of other DENN-domain-containing proteins such as the Rab35 GEF [14]. Crag exhibits GEF activity against Rab10 with much faster kinetics than against Rab11, indicating that the slow kinetics may be due to properties of Rab11. This is further supported by the slow kinetics of EDTA that triggers GDP release of Rab11. It's possible that the GDP/GTP exchange of Rab11 requires other co-factors besides its GEF, as, for example, documented for Rab6 [61],[62].

CaM is a ubiquitously expressed calcium sensor [63]. In the *Drosphila* photoreceptor cells, photoactivation leads to influx of Ca^2+^ and activation of CaM. It has been shown that CaM is required for the termination of the photoresponse in several steps, including TRP inactivation and conformational change of metaRh [59],[64]. Crag contains a CaM binding site and interacts with CaM in a calcium-dependent manner [7],[8]. In our in vitro GEF assay, the presence of CaM and Ca^2+^ indeed enhances the GEF activity of Crag. Hence, it is possible that a light-induced increase of intracellular Ca^2+^ level enhances Crag activity via CaM binding. The activation of Crag/Rab11 then may serve to replenish rhabdomeric Rh1, whose loss is also induced by light stimulation.

### DENND4A and Human Photoreceptor Degeneration

In vertebrate rod cells, polarized transport of Rh is mediated by post-Golgi vesicles that bud from the TGN and fuse with the base of the outer segment [65],[66]. Rab11 has been detected on rhodopsin-bearing post-Golgi vesicles in photoreceptors [67],[68]; however, it has not yet been shown that Rab11 is required for Rh trafficking. DENND4 proteins are highly similar to Crag. Here we showed that expression of the UAS–human DENND4A construct not only rescues the lethality but also rescues the light-induced photoreceptor degeneration caused by loss of *Crag* (Figure S9), showing that the molecular function of DENND4A is also conserved. Moreover, three different subtypes of Usher syndrome, an inherited condition characterized by hearing loss and progressive vision loss, have been mapped to the vicinity of the DENND4A locus at 15q22.31 [69]–[71]. Hence, DENND4A may also function through Rab11 in human photoreceptors, and loss of DENND4A may lead to photoreceptor degeneration.

## Materials and Methods

### Fly Strains and Genetics

#### Mutagenesis

∼8,600 *y w P{neoFRT}19A^isogenized^* male flies were treated with low concentration of ethylmethane sulfonate (7.5–15 mM) to induce mutations. Flies were starved for 12 h followed by feeding with ethylmethane sulfonate–laced sucrose solution for 15 h. The treated males were crossed with *Df(1)JA27/FM7c, Kr-GAL4 UAS-GFP* (hereafter abbreviated as *Kr-GFP*) females. The F1 females y w mut* P{neoFRT}19A/FM7c, Kr-GFP were individually crossed with *FM7c, Kr-GFP/Y* males to establish stocks and then screened for lethal mutations. 5,859 stocks bearing lethal mutations were established. Female y w mut* P{neoFRT}19A/FM7c, Kr-GFP flies from these stocks were crossed with *cl(1) P{neoFRT}19A/Dp(1;Y)y^+^ v^+^; ey-FLP* males to generate flies with homozygous mutant clones in the eye. These flies were aged for 3 wk, and ERGs were performed on the mutant patches marked by a loss of *w+*, as described previously [38].

#### Mapping of the *XE10* group

Female y w mut* P{neoFRT}19A/FM7c, Kr-GFP flies were crossed to a set of large duplications (∼1–2 Mb) covering the X chromosome [39] to roughly map the lethality and generate rescued males. Mutations rescued by the same duplication were then crossed inter se to establish complementation groups. For the *XE10* group, the alleles were rescued by *Dp(1;Y)619* (7D–8B3). We then crossed the *XE10* alleles with deficiencies within this region, and found that *Df(1)BSC627* and *Df(1)BSC592* fail to complement all *XE10* alleles. These Dfs share a ∼120-kb interval, and a insertion *PBac{WH}CG12659^f07899^* [41] in CG12737 fails to complement all *XE10* alleles. CG12737 corresponds to *Crag*, and a null *Crag^CJ101^* allele was obtained from Trudi Schupbach [8]. *Crag^CJ101^* fails to complement all *XE10* alleles.

For ERG, Chaoptin staining, and TEM experiments, we crossed *y w Crag P{neoFRT}19A/FM7c, Kr-GFP* females with *cl(1) P{neoFRT}19A/Dp(1;Y)y^+^ v^+^ (3); ey-FLP* males. Mutant clones correspond to white patches. Flies in which more than 95% of the eyes is mutant were typically analyzed. For controls, homozygous *y w P{neoFRT}19A^iso^* females were crossed with the *cl(1) P{neoFRT}19A/Dp(1;Y)y^+^ v^+^ (3); ey-FLP* males to produce clones. For immunostaining in the L3 larval eye imaginal discs, *y w Crag^C^ P{neoFRT}19A/FM7c, Kr-GFP* female flies were crossed with *cl(1) Ubi-GFP P{neoFRT}19A/Dp(1;Y)y^+^ v^+^ (3); ey-FLP* males, and L3 female larva that lacked Kr-GFP expression were selected. For immunostaining in photoreceptors and the blue light experiments, *y w cl(1) P{neoFRT}19A/Dp(1;Y)y^+^ v^+^ (3); ey-FLP* males were used to eliminate pigment from the eye. Mutant clones can be recognized, as they exhibit a subtle roughness. To express Rab11-CA in *Crag* clones, *y w Crag P{neoFRT}19A/FM7c; P{UASp-YFP.Rab11.Q70L}^CG13895^* females were crossed with *P{GMR-hid}, y w P{neoFRT}19A/Y; P{GAL4-ey.H}, P{UAS-FLP1.D}, P {rh1-GAL4}* males. For controls, *y w Crag P{neoFRT}19A/FM7c; P{UAS-GFP}* and *y w P{neoFRT}19A^iso^; P{UAS-GFP}* females were crossed with the same males.

### Electroretinograms

ERG recordings were performed as previously described [38]. In brief, adult flies were glued to a glass slide, a recording probe was placed on the surface of the eye, and a reference probe was inserted in the thorax. A 1-s flash of white light was given, and the response was recorded.

### Single-Photoreceptor Recording

Single-photoreceptor recordings were performed as previously described [72]. In brief, a small hole was cut on the cornea of the eye, and the hole was sealed by Vaseline. A reference probe was placed at the back of the head, and a recording probe was inserted into the retina through the previously cut hole. The membrane potential was monitored by AXOCLAMP-2B (Axon Instruments). When the membrane potential dropped to below −60 mV, the fly was given a 10-ms white light stimulation (white LED, 7,000 mcd, super bright LEDs), and the response was recorded.

### Transmission Electron Microscopy

Electron microscopy of the photoreceptor and the lamina was performed as described [73]. In brief, fly heads were dissected and fixed at 4°C in 4% paraformaldehyde, 2% glutaraldehyde, 0.1 M sodium cacodylate, and 0.005% CaCl_2_ (pH 7.2) overnight, postfixed in 1% OsO_4_ for 1 h, dehydrated in ethanol and propylene oxide, and then embedded in Embed-812 resin (Electron Microscopy Sciences). Thin sections (∼50 nm) of the photoreceptor and lamina were stained in 4% uranyl acetate and 2.5% lead nitrate, and TEM images were captured using a transmission electron microscope (model 1010, JEOL) with a digital camera (US1000, Gatan). For quantification, the sizes of rhabdomeres are determined in Image J.

### Immunostaining of the Photoreceptor Cells

For cross-sections, fly heads were bisected, fixed in 4% paraformaldehyde for 3 h, dehydrated in acetone, embedded in LR white resin (Polysciences), and sectioned. Immunostaining was performed as described by [51]. For whole mount staining of fly heads, heads were fixed in 4% formaldehyde upon removal of the proboscis. The photoreceptors were dissected and fixed for 15 min. Standard immunostaining procedures were then performed, as previously described [74]. Images were obtained with a Zeiss LSM 710 confocal microscope. Antibodies were as follows: rat anti-Crag [8], 1∶200; mouse monoclonal anti-Rab11 (BD Biosciences), 1∶20; rabbit anti-Rab11 [34], 1∶1,000; mouse anti-Rh1 [75], 1∶50; rabbit anti-TRP [76], 1∶100; rabbit anti-InaD [45], 1∶200; rabbit anti-Arr2 [52], 1∶200; biotin-conjugated Peanut agglutinin (Vector Labs), 1∶1,000; Alexa 488–conjugated phalloidin (Invitrogen), 1∶200; and Alexa 405–, Alexa 488–, Cy3-, or Cy5-conjugated secondary antibodies (Jackson ImmunoResearch), 1∶200.

### Blue Light Treatment and Rh1 Detection

Flies were kept in a box with a blue LED (465 nm) light panel (∼1,000 lux) for 6 h with or without 24 h recovery in dim white light. For Western blots, fly heads were separated, homogenized, and incubated with SDS loading buffer (50 mM Tris-HCl [pH 6.8], 2% SDS, 10% glycerol, 1% β-mercaptoethanol, 12.5 mM EDTA, and 0.02% bromophenol blue) for 20 min at room temperature. For dot blots, fly heads were homogenized in SDS buffer with 1 M urea, as previously described [36]. Sample buffer was applied to natural cellulose membrane and air dried, followed by antibody detection. A polyclonal rabbit anti-Rh1 antibody (1∶2,000) was used to detect Rh1 [34].

### S2 Cell Transfection, Immunostaining, and Co-Immunoprecipitation

S2 cells were cultured in Schneider's media with 10% fetal bovine serum at room temperature and transfected using Lipofectamin LTX (Invitrogen). For immunostaining, mouse anti-FLAG (M2, Sigma) and rat anti-HA (Roche) antibodies were used to detect Crag (FLAG) and Rab (HA) proteins. For co-IP, cells were harvested 40 h after infection and lysed with lysis buffer (50 mM Tris-HCl [pH 8.0], 100 mM NaCl, 1 mM EDTA, 1% Nonidet P-40, and Complete Protease Inhibitor Cocktail Tablets [Roche]). For comparing the binding affinity of Rab11-DN and Rab11-CA with Crag, EDTA was removed from the lysis buffer. Then the cell lysates were incubated with anti-HA affinity gel (Sigma) for 3 h in lysis buffer at 4°C. The anti-HA gel was pelleted and analyzed by Western blot using anti-HA (Roche), 1∶2,000, or anti-FLAG (M2, Sigma), 1∶1,000, followed by HRP-conjugated secondary antibodies (Jackson ImmunoResearch), 1∶5,000.

### Protein Expression and Purification

*Crag* cDNA tagged with GST (GE Healthcare) was cloned into a pOPINJ vector [77]. The construct was transfected into SF9 cells (Invitrogen), along with Bsu36I-digested BacPAK6 DNA. Recombined viruses were harvested from the medium and amplified with a second round of infection. To express Crag protein, Hi-5 cells (Invitrogen) were infected with the virus and cultured for 40 h before they were harvested and lysed. Crag protein was then purified from the cell lysates with glutathione sepharose 4B (GE Healthcare). Rab5, Rab10, Rab11, and CaM proteins were generated using the same protocol.

### GEF Assay

GEF assays were performed as described previously [61]. For preloading with GDP, Rab5, Rab10, and Rab11 were incubated in 110 mM NaCl, 50 mM Tris-HCl (pH 8.0), 1 mM EDTA, 0.8 mM DTT, 0.005% Triton X-100, and 50 µM BODIPY-GDP (Invitrogen) for 60 min at 30°C. 10 mM MgCl_2_ was then added to stop the reaction. 0.1 uM preloaded Rab proteins were then incubated with or without 0.05 µM Crag in a 110 mM NaCl, 50 mM Tris-HCl (pH 8.0), 12 mM MgCl_2_, 0.8 mM DTT, and 2 mM GDP solution. To test whether CaM regulates Crag function, 0.05 µM CaM and 1 mM CaCl_2_ were added to the above reaction. As a positive control, preloaded Rab11 was incubated with 10 mM EDTA in the above solution. The fluorescence intensity was recorded automatically by a FLUOstar OPTIMA plate reader (BMG Labtech) every 30 s over a 2-h period.

## Supporting Information

Figure S1 ***Crag*** **mutant photoreceptors target their axons and synapses properly to the medulla, but exhibit subtle defects in ommatidial organization.** *Crag* mutant R7 and R8 photoreceptors target properly to the medulla. *y w FRT19A^iso^* (control), *y w Crag^A^ P{neoFRT}19A/FM7c, Kr-GFP*, or *y w Crag^C^ P{neoFRT}19A/FM7c, Kr-GFP* female flies were crossed with *cl(1) P{neoFRT}19A/Dp(1;Y)y^+^ v^+^ (3); ey-FLP* males. Progenies with large mutant eye patches (>95%) were assayed. 1-d-old fly brains were dissected and stained with Chaoptin antibody (mAb24B10, Developmental Studies Hybridoma Bank). Arrows point to the R7 terminals, and arrowheads point to R8 terminals. No significant differences between the two *Crag* alleles and the controls were observed.
(TIF)

Figure S2 ***Crag*** **mutant R1–R6 photoreceptors target properly to the lamina, but the terminal portions of the photoreceptors are affected in aged flies upon light exposure.** (A and B) TEM of lamina sections in 1-d-old flies of control (*y w FRT19A^iso^*) and *Crag^C^* mutant clones shows that *Crag* mutant R1–R6 photoreceptors target properly to the lamina. Each photoreceptor terminal is outlined by a red circle. Typical terminal structures were observed in *Crag* mutant clones, including normal capitate projections, active zones, synaptic vesicles, and mitochondria. Scale bar, 1 µM. (C and D) Average number of terminals per cartridge (C) and average number of capitate projections per terminal (D) were calculated, *n* = 10. There are no significant differences between control and *Crag* alleles. (E and F) TEM of lamina cross-sections in flies cultured for 2 wk with 12-h on/off light exposure. Red cycles outline the wild-type R1–R6 photoreceptor terminals in (E). However, in *Crag* mutants, the intracellular structures of photoreceptor terminals are not recognizable (red arrows in [F]).
(TIF)

Figure S3 **Phototransduction is not affected in dark-raised** ***Crag*** **mutant photoreceptors.** (A) Representative pictures of control mosaic eyes used for ERG experiments. *y w FRT19A^iso^* females were crossed with *cl(1) P{neoFRT}19A/Dp(1;Y)y^+^ v^+^ (3); ey-FLP* males to generate homozygous clones in the eye. *y w FRT19A^iso^* photoreceptors are marked by white patches. (B) Representative pictures of mosaic eyes that contain homozygous *Crag^C^* mutant photoreceptors, which are marked by white patches. The corneas of the eyes with *Crag^C^* clones show some roughness; however, the function of these photoreceptor cells is not affected, as determined by ERG and single-cell recordings. (C) Intracellular recordings of single photoreceptors in response to a brief light stimulus (10 ms). 2-d-old flies raised in the dark were used for the experiments. Each trace is the average of 20 repetitive recordings of the same photoreceptor. Note that control and *Crag^C^* mutant photoreceptors exhibit similar responses to light. (D and E) Average depolarization amplitude (D) and average decay time (E) were calculated for the single-photoreceptor recordings, *n* = 6. The decay time was measured from the peak to the 80% repolarization point. There are no significant differences between control and *Crag* alleles.
(TIF)

Figure S4 **Rh1 levels gradually decrease in** ***Crag*** **mutant clones in flies raised in light/dark cycle.** Fly heads with control or *Crag^C^* mutant clones in the eyes were dissected from flies that were kept in the dark or in light/dark cycle at different stages. Western blots were performed to detect total Rh1 and Actin (loading control) levels. In *Crag* mutant clones, the Rh1 levels gradually decrease when the flies are aged in light/dark cycle but not when they are kept in the dark.
(TIF)

Figure S5 **Blue light triggers a much more severe internalization of Rh1 than white light, and accumulation of Rh1 in** ***Crag*** **mutant photoreceptors is not due to PDA.** (A) Western blot of Rh1 and Actin of fly heads dissected from flies that were kept in the dark or after 6 h in white light. Note that the Rh1 levels are not significantly altered after 6 h of white light exposure. (B) Whole mount immunostaining of Rh1 in control and *Crag^C^* mutant photoreceptors exposed to 6 h of white light. Internalization of Rh1 is not obvious in both genotypes when compared to blue-light-triggered endocytosis of Rh1 (see Figure 4C). (C) Whole mount immunostaining of Rh1 in control and *Crag^C^* mutant photoreceptors kept for 6 h in blue light, 20 min in orange light, and 18 h in the dark. Orange light exposure was used to terminate the PDA caused by blue light exposure. Note that Rh1 accumulates in the cytosol in this paradigm.
(TIF)

Figure S6 **TEM of photoreceptor cross-sections of flies exposed to blue light.** TEM analyses of control and *Crag^C^* mutant photoreceptors kept in the dark, exposed to 6 h blue light, or exposed to 6 h blue light with 18 or 36 h recovery in the dark. Note that the electron density of the cytosol correlates with the Rh1 levels in the cytosol (see Figure 4C for immunostaining results). Also note that the rhabdomeres start to break down after 6 h of blue light stimulation and 36 h of dark incubation in *Crag^C^* mutant photoreceptors. Scale bar, 2 µM.
(TIF)

Figure S7 **Screen for potential targets of Crag: the Drosophila Rab proteins.** 31 DN Rab transgenes were examined. *Rh1-GAL4* was used to drive their expression in the adult eye. ERGs were performed at day 1 and after 21 d of culture in a light/dark cycle or in complete darkness. Expression of Rab1-DN and Rab5-DN leads to a reduction of ERG amplitude in light/dark and dark conditions. Expression of Rab11-DN leads to a reduction of ERG amplitude only when flies are treated with light. No significant differences were observed when other DN-Rabs were expressed in either light condition in our screen.
(TIF)

Figure S8 **Mutations in** ***Crag*** **and** ***Rab11*** **lead to a similar time course of photoreceptor degeneration.** (A) Representative ERG traces at different stages in the light/dark cycle of flies of the following genotypes: *Rh1-GAL4, UAS-GFP* (control); *Crag* mutant clones with *Rh1-GAL4, UAS-GFP* (*Crag^C^*+GFP); *Rh1-GAL4, UAS-Rab11-S25N* (Rab11-DN); and *Crag* mutant clones with *Rh1-GAL4, UAS-Rab11-S25N* (*Crag^C^*+*Rab11-DN*). Note that the ERG amplitudes are gradually decreased when Crag and/or Rab11 function is impaired. (B) Quantification of the ERG depolarization amplitudes shown in (A). Ten ERG traces were measured for each genotype at each time point. Note that when Crag and Rab11 function are both impaired, the degeneration rate of the photoreceptors is not significantly enhanced.
(TIF)

Figure S9 **Expression of DENND4A, the human homolog of** ***Crag*** **, rescues the ERG defects and photoreceptor degeneration associated with Crag loss of function.** (A) ERG traces of wild-type controls (*y w FRT19A^iso^*), *Crag* mutants, and *Crag* mutant flies rescued by expression of DENND4A with *da-GAL4*. These flies were cultured in a 12-h on/off light cycle for 14 d. Consistent with previous data, the ERG depolarization amplitude is reduced in *Crag* mutants but is unaffected in both wild-type controls and *Crag* mutant flies rescued by expression of DENND4A. (B) Quantification of the ERG depolarization amplitudes shown in (A). Ten ERG traces were measured for each genotype. (C) TEM of photoreceptor cross-sections of *Crag* mutant cells with or without DENND4A expression upon 14 d of light/dark exposure. Rhabdomere structures are severely disrupted in *Crag* mutant cells but not in *Crag* mutant cells expressing DENND4A. Scale bar, 1 µM. (D) Quantification of the rhabdomere areas shown in (C). Rhabdomere area is significantly smaller in *Crag* mutant photoreceptors than in *Crag* mutant photoreceptors expressing DENND4A. Ten ommatidia from different cross-sections were analyzed for each genotype.
(TIF)

Figure S10 **Model of Crag function.** (A) Photoactivation. Rh1 undergoes a conformational change to metaRh upon absorption of a photon (480 nm). MetaRh in turn signals through a G-protein-coupled cascade and triggers the opening of TRP channels and the influx of Ca^2+^ and Na^+^ into photoreceptor cells. (B) Upon light activation, the majority of metaRh is converted back into Rh1 on rhabdomeres by exposure to another photon (580 nm). However, a subpopulation of metaRh is internalized and degraded through a lysosomal pathway. (C) Light induces Ca^2+^ influx and activates CaM, which in turn promotes Crag activity. Crag activates Rab11 as a GEF, and Rab11 is required to transport newly synthesized Rh1 to the rhabdomeres to maintain the rhabdomeric Rh1 level. (D) In the absence of Crag, internalization and degradation of metaRh is unaffected, whereas trafficking of Rh1 to the rhabdomeres is impaired. Therefore, loss of *Crag* leads to accumulation of Rh1 in the cytosol, shrinkage of rhabdomeres, and, eventually, photoreceptor degeneration.
(TIF)

## Abbreviations

CA: constitutively active
co-IP: co-immunoprecipitation
DN: dominant negative
ERG: electroretinogram
GEF: guanine nucleotide exchange factor
metaRH: metarhodopsin
PDA: prolonged depolarizing afterpotential
Rh: Rhodopsin
TEM: transmission electron microscopy
TGN: trans-Golgi network
TRP: transient receptor potential
