# Supplementary figures and images for: Crag Is a GEF for Rab11 Required for Rhodopsin Trafficking and Maintenance of Adult Photoreceptor Cells
Source: PLoS Biol. 2012 Dec 4;10(12):e1001438. doi: 10.1371/journal.pbio.1001438 (PMC3514319; doi:10.1371/journal.pbio.1001438)

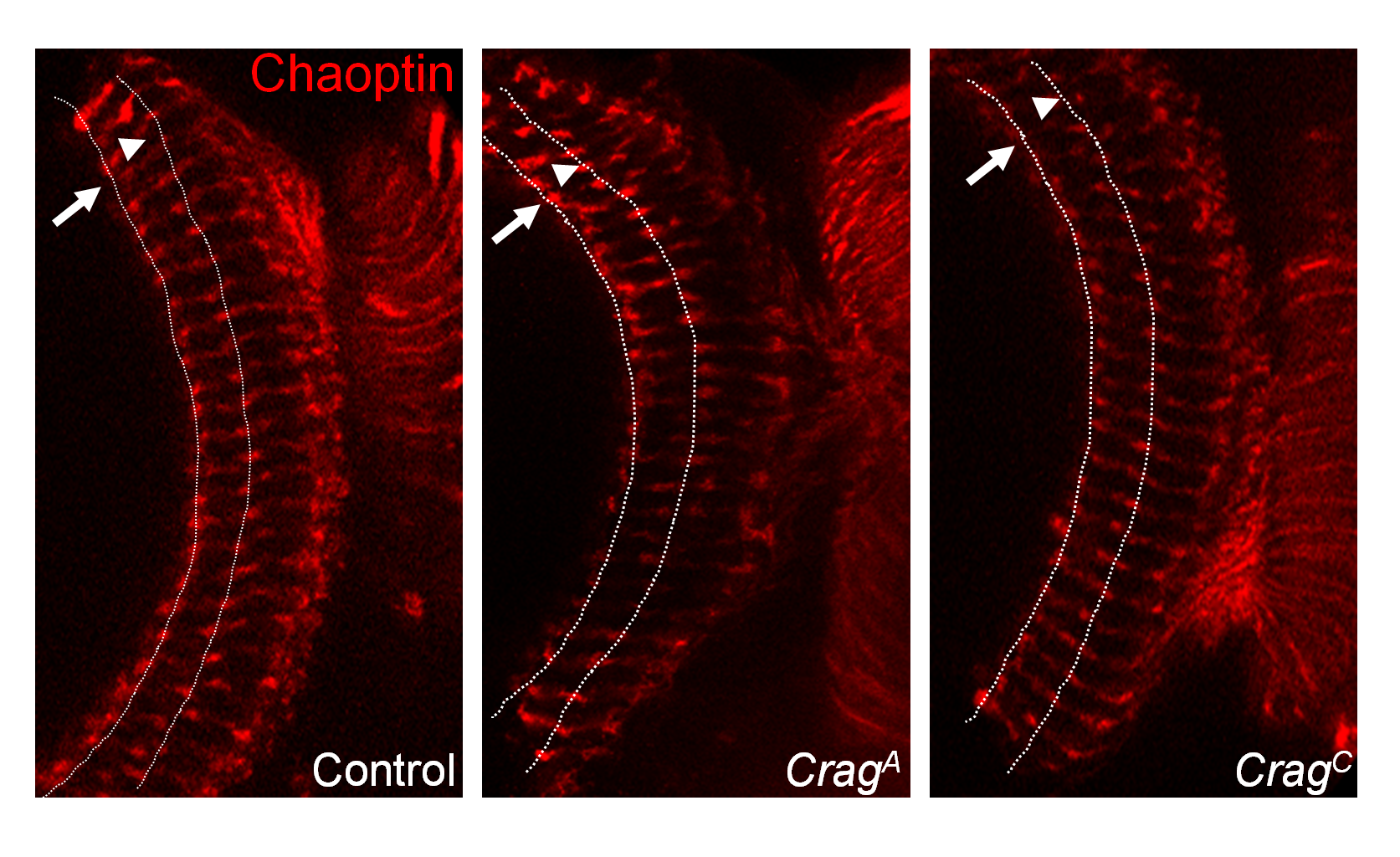

Supplement: Figure S1 — Crag mutant photoreceptors target their axons and synapses properly to the medulla, but exhibit subtle defects in ommatidial organization. Crag mutant R7 and R8 photoreceptors target properly to the medulla. y w FRT19Aiso (control), y w CragA P{neoFRT}19A/FM7c, Kr-GFP, or y w CragC P{neoFRT}19A/FM7c, Kr-GFP female flies were crossed with cl(1) P{neoFRT}19A/Dp(1;Y)y+ v+ (3); ey-FLP males. Progenies with large mutant eye patches (>95%) were assayed. 1-d-old fly brains were dissected and stained with Chaoptin antibody (mAb24B10, Developmental Studies Hybridoma Bank). Arrows point to the R7 terminals, and arrowheads point to R8 terminals. No significant differences between the two Crag alleles and the controls were observed. (TIF) [file pbio.1001438.s001.tif]

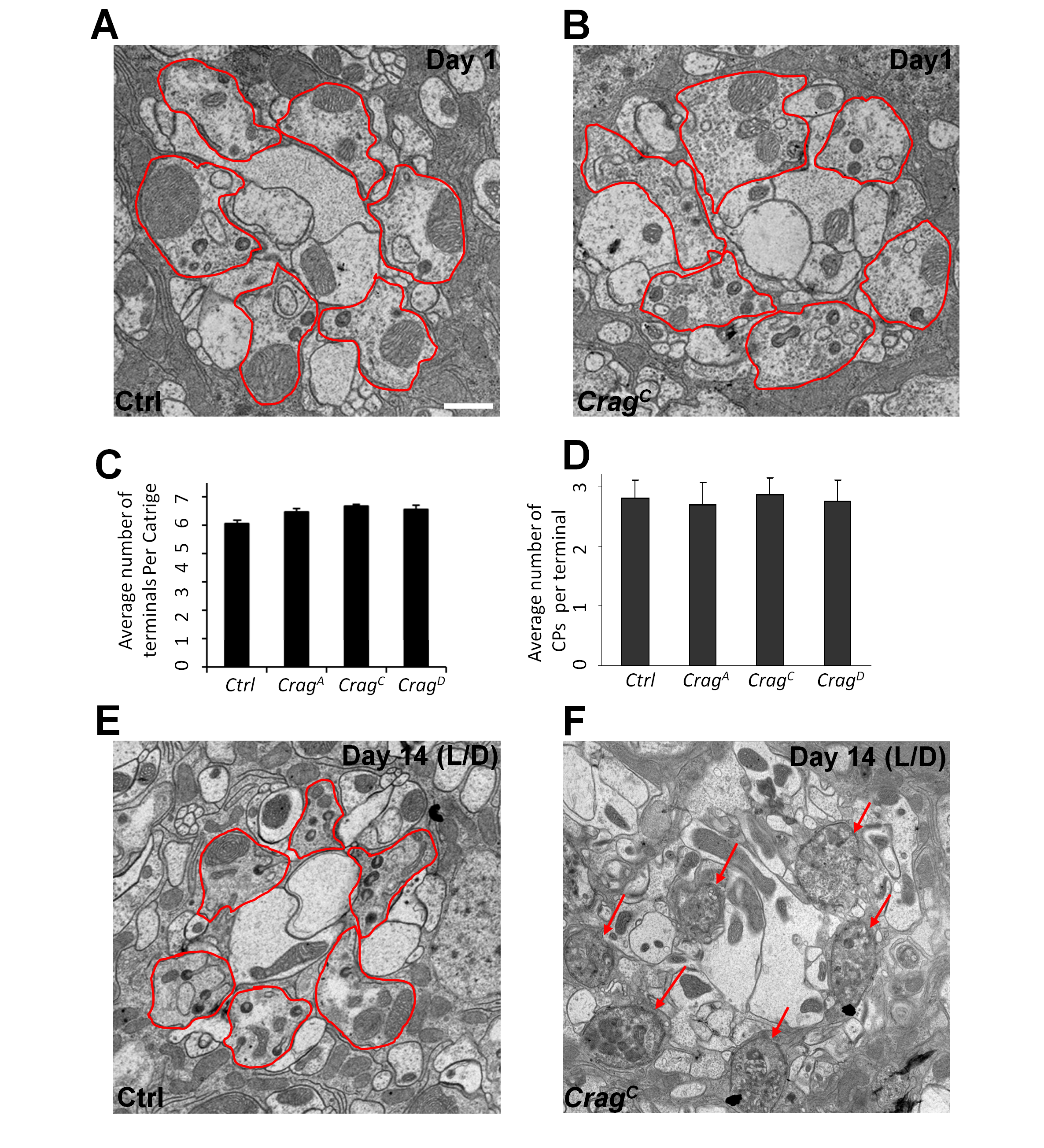

Supplement: Figure S2 — Crag mutant R1–R6 photoreceptors target properly to the lamina, but the terminal portions of the photoreceptors are affected in aged flies upon light exposure. (A and B) TEM of lamina sections in 1-d-old flies of control (y w FRT19Aiso) and CragC mutant clones shows that Crag mutant R1–R6 photoreceptors target properly to the lamina. Each photoreceptor terminal is outlined by a red circle. Typical terminal structures were observed in Crag mutant clones, including normal capitate projections, active zones, synaptic vesicles, and mitochondria. Scale bar, 1 µM. (C and D) Average number of terminals per cartridge (C) and average number of capitate projections per terminal (D) were calculated, n = 10. There are no significant differences between control and Crag alleles. (E and F) TEM of lamina cross-sections in flies cultured for 2 wk with 12-h on/off light exposure. Red cycles outline the wild-type R1–R6 photoreceptor terminals in (E). However, in Crag mutants, the intracellular structures of photoreceptor terminals are not recognizable (red arrows in [F]). (TIF) [file pbio.1001438.s002.tif]

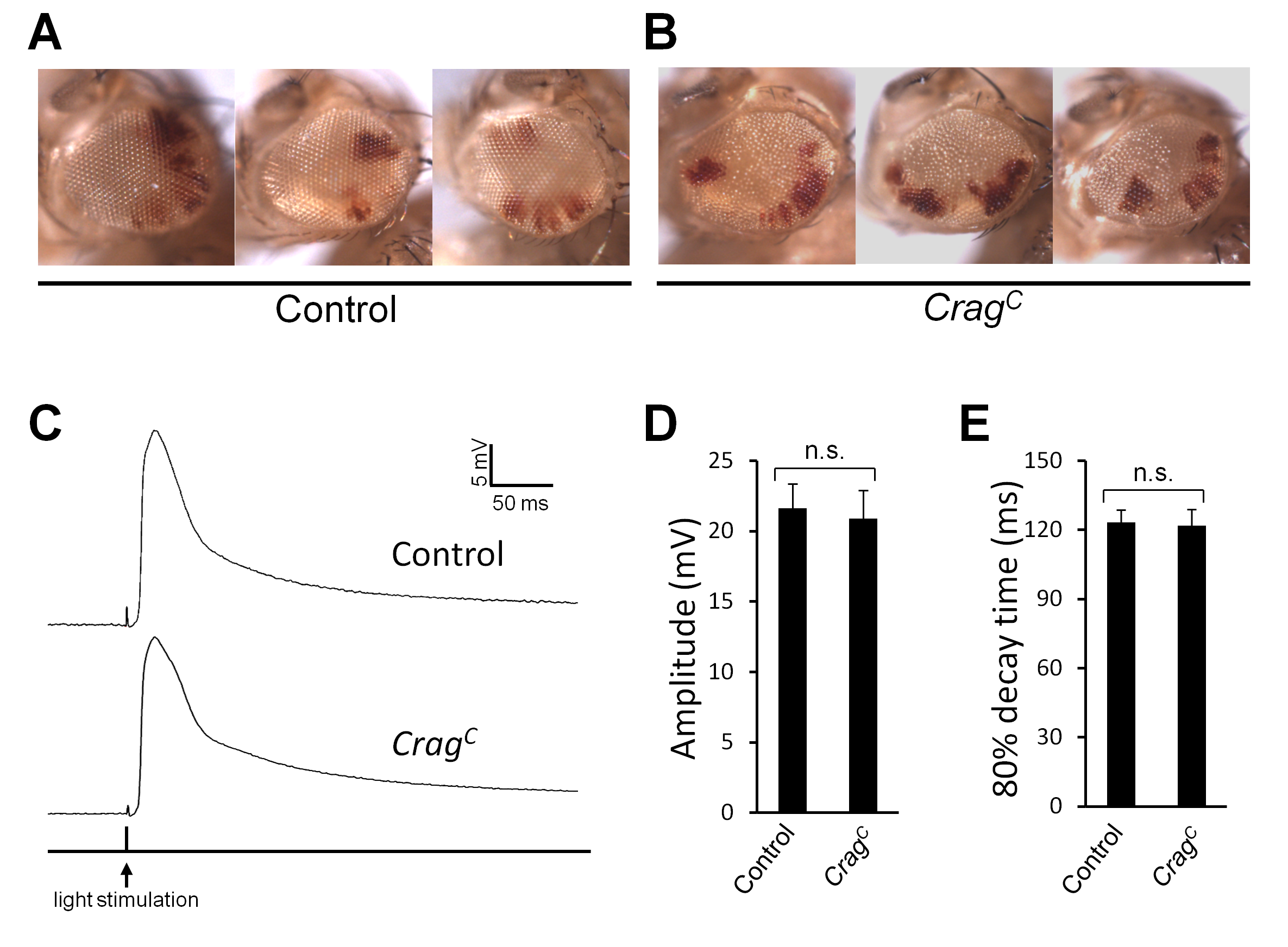

Supplement: Figure S3 — Phototransduction is not affected in dark-raised Crag mutant photoreceptors. (A) Representative pictures of control mosaic eyes used for ERG experiments. y w FRT19Aiso females were crossed with cl(1) P{neoFRT}19A/Dp(1;Y)y+ v+ (3); ey-FLP males to generate homozygous clones in the eye. y w FRT19Aiso photoreceptors are marked by white patches. (B) Representative pictures of mosaic eyes that contain homozygous CragC mutant photoreceptors, which are marked by white patches. The corneas of the eyes with CragC clones show some roughness; however, the function of these photoreceptor cells is not affected, as determined by ERG and single-cell recordings. (C) Intracellular recordings of single photoreceptors in response to a brief light stimulus (10 ms). 2-d-old flies raised in the dark were used for the experiments. Each trace is the average of 20 repetitive recordings of the same photoreceptor. Note that control and CragC mutant photoreceptors exhibit similar responses to light. (D and E) Average depolarization amplitude (D) and average decay time (E) were calculated for the single-photoreceptor recordings, n = 6. The decay time was measured from the peak to the 80% repolarization point. There are no significant differences between control and Crag alleles. (TIF) [file pbio.1001438.s003.tif]

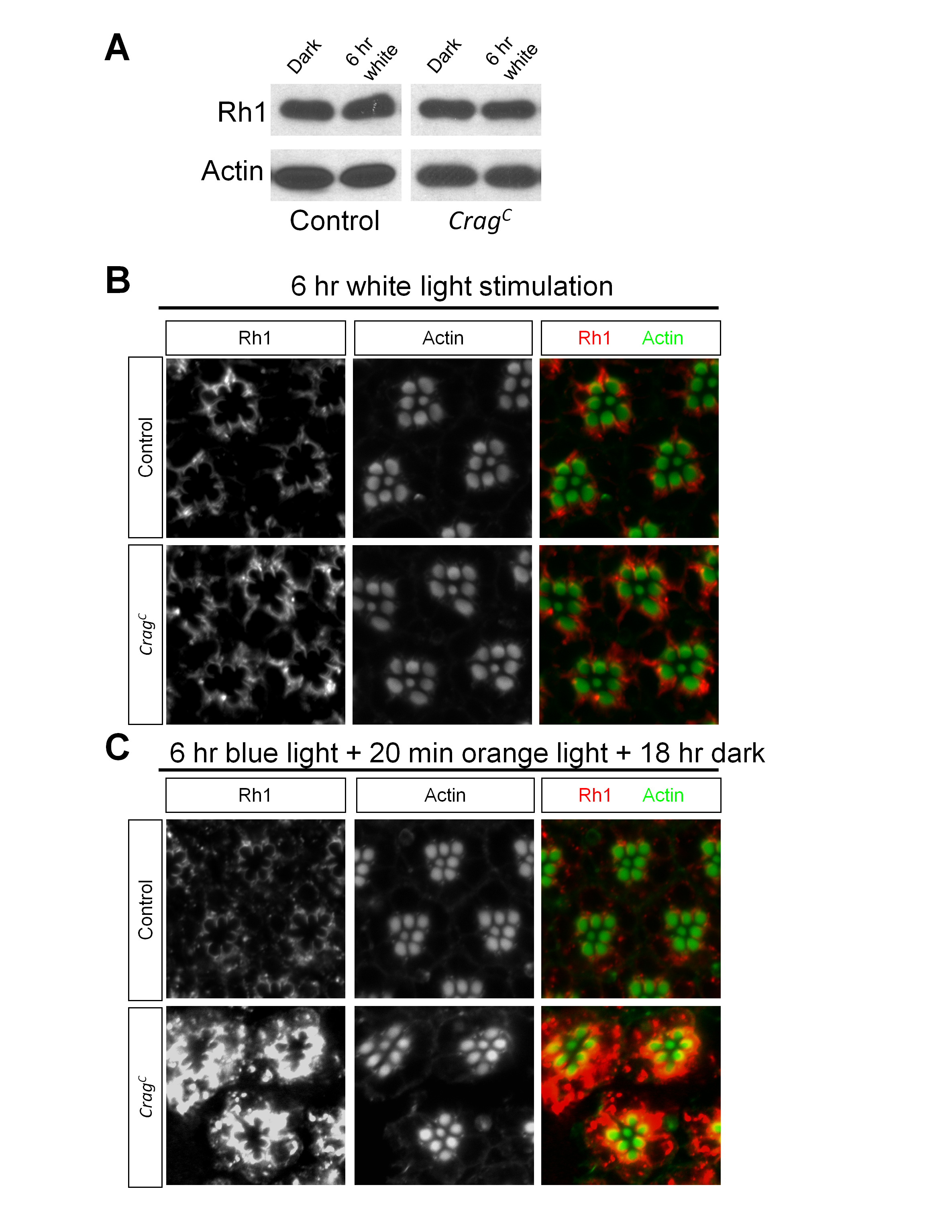

Supplement: Figure S5 — Blue light triggers a much more severe internalization of Rh1 than white light, and accumulation of Rh1 in Crag mutant photoreceptors is not due to PDA. (A) Western blot of Rh1 and Actin of fly heads dissected from flies that were kept in the dark or after 6 h in white light. Note that the Rh1 levels are not significantly altered after 6 h of white light exposure. (B) Whole mount immunostaining of Rh1 in control and CragC mutant photoreceptors exposed to 6 h of white light. Internalization of Rh1 is not obvious in both genotypes when compared to blue-light-triggered endocytosis of Rh1 (see Figure 4C). (C) Whole mount immunostaining of Rh1 in control and CragC mutant photoreceptors kept for 6 h in blue light, 20 min in orange light, and 18 h in the dark. Orange light exposure was used to terminate the PDA caused by blue light exposure. Note that Rh1 accumulates in the cytosol in this paradigm. (TIF) [file pbio.1001438.s005.tif]

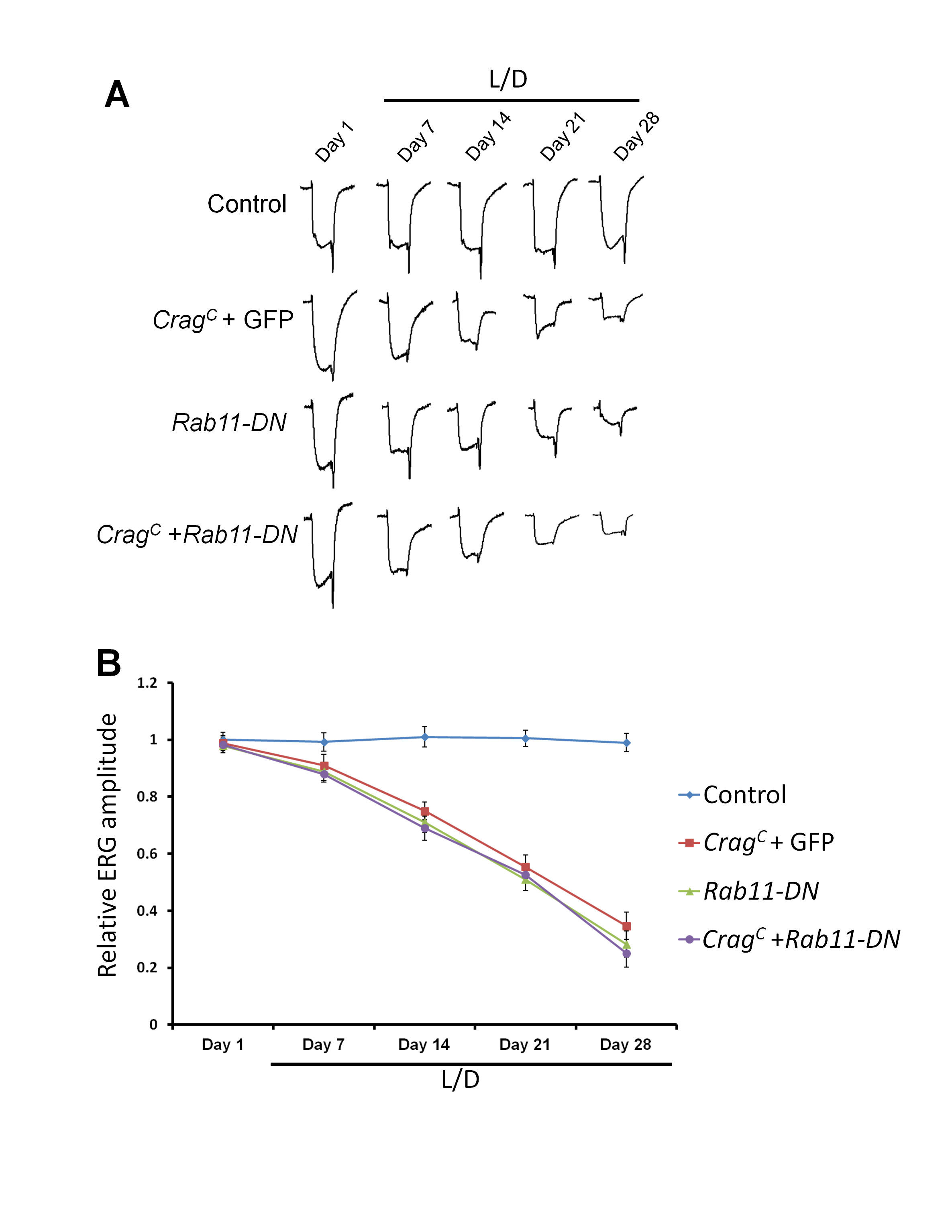

Supplement: Figure S8 — Mutations in Crag and Rab11 lead to a similar time course of photoreceptor degeneration. (A) Representative ERG traces at different stages in the light/dark cycle of flies of the following genotypes: Rh1-GAL4, UAS-GFP (control); Crag mutant clones with Rh1-GAL4, UAS-GFP (CragC+GFP); Rh1-GAL4, UAS-Rab11-S25N (Rab11-DN); and Crag mutant clones with Rh1-GAL4, UAS-Rab11-S25N (CragC+Rab11-DN). Note that the ERG amplitudes are gradually decreased when Crag and/or Rab11 function is impaired. (B) Quantification of the ERG depolarization amplitudes shown in (A). Ten ERG traces were measured for each genotype at each time point. Note that when Crag and Rab11 function are both impaired, the degeneration rate of the photoreceptors is not significantly enhanced. (TIF) [file pbio.1001438.s008.tif]

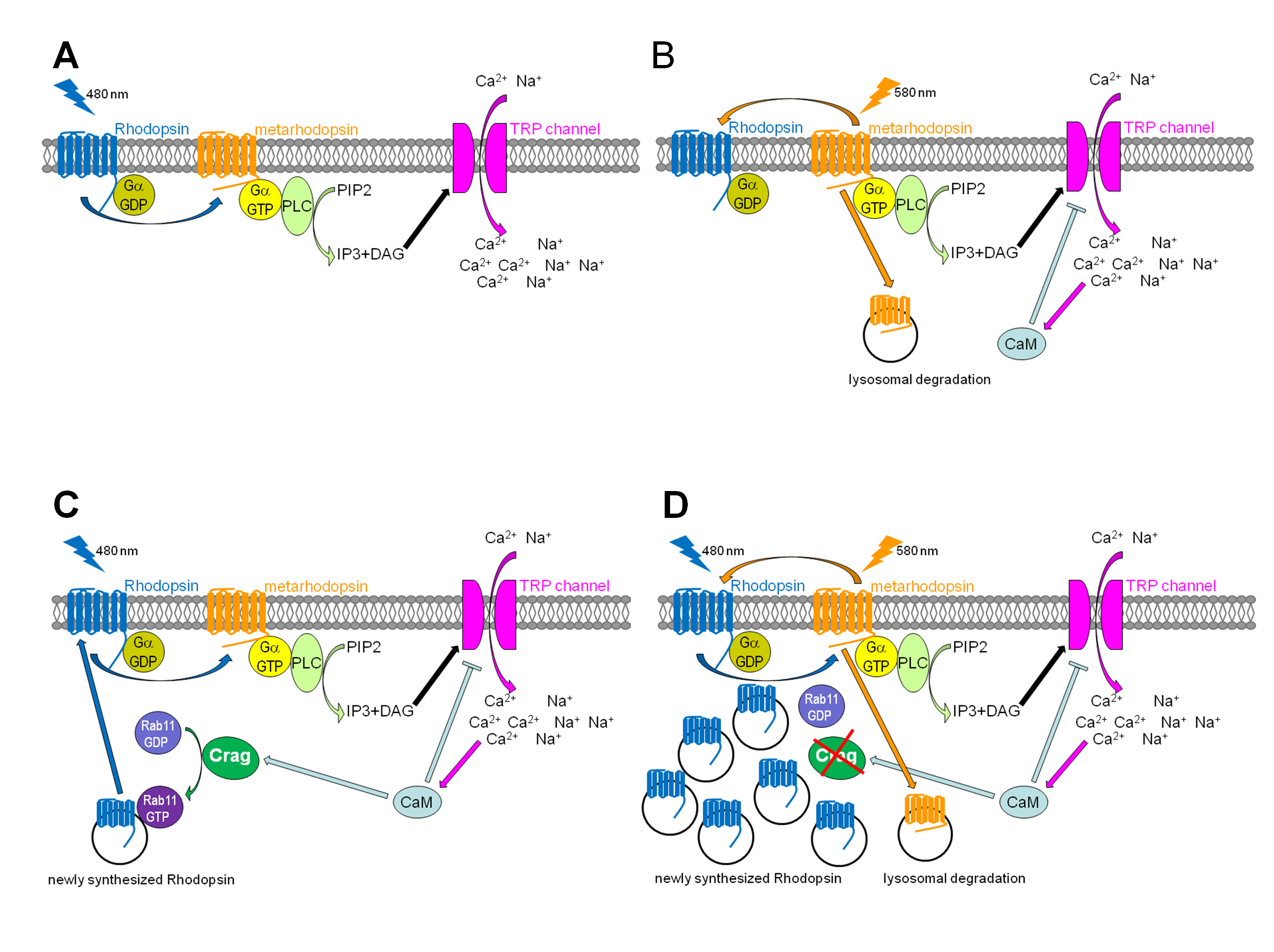

Supplement: Figure S10 — Model of Crag function. (A) Photoactivation. Rh1 undergoes a conformational change to metaRh upon absorption of a photon (480 nm). MetaRh in turn signals through a G-protein-coupled cascade and triggers the opening of TRP channels and the influx of Ca2+ and Na+ into photoreceptor cells. (B) Upon light activation, the majority of metaRh is converted back into Rh1 on rhabdomeres by exposure to another photon (580 nm). However, a subpopulation of metaRh is internalized and degraded through a lysosomal pathway. (C) Light induces Ca2+ influx and activates CaM, which in turn promotes Crag activity. Crag activates Rab11 as a GEF, and Rab11 is required to transport newly synthesized Rh1 to the rhabdomeres to maintain the rhabdomeric Rh1 level. (D) In the absence of Crag, internalization and degradation of metaRh is unaffected, whereas trafficking of Rh1 to the rhabdomeres is impaired. Therefore, loss of Crag leads to accumulation of Rh1 in the cytosol, shrinkage of rhabdomeres, and, eventually, photoreceptor degeneration. (TIF) [file pbio.1001438.s010.tif]
